# The Extraordinary Diversity of *Merodon avidus* Complex (Diptera: Syrphidae)—Adding New Areas, New Species and a New Molecular Marker

**DOI:** 10.3390/insects15020105

**Published:** 2024-02-02

**Authors:** Ante Vujić, Nataša Kočiš Tubić, Snežana Radenković, Jelena Ačanski, Laura Likov, Maja Arok, Iva Gorše, Mihajla Djan

**Affiliations:** 1 Department of Biology and Ecology, Faculty of Sciences, University of Novi Sad, Trg Dositeja Obradovića 2, 21000 Novi Sad, Serbia; ante.vujic@dbe.uns.ac.rs (A.V.); snezana.radenkovic@dbe.uns.ac.rs (S.R.); laura.likov@dbe.uns.ac.rs (L.L.); iva.gorse@dbe.uns.ac.rs (I.G.); mihajla.djan@dbe.uns.ac.rs (M.D.); 2 BioSense Institute, University of Novi Sad, Dr Zorana Ðinđića 1, 21000 Novi Sad, Serbia; acanski@biosense.rs (J.A.); maja.arok@biosense.rs (M.A.)

**Keywords:** COI gene, 28S rRNA gene, distribution, hoverflies, taxonomy, wing shape

## Abstract

**Simple Summary:**

In this paper, the *Merodon avidus* (Diptera, Syrphidae) species complex was revised, whereupon we discovered and described four new species for science. An integrative taxonomy approach was used to delimit species boundaries. The molecular analysis, the differences in the shape of the wings, the morphological characters of the adults and the distribution ranges successfully separated all species from the complex.

**Abstract:**

In this paper, the *Merodon avidus* (Diptera, Syrphidae) species complex was revised, whereupon we discovered and described four new species for science: *Merodon atroavidus* Vujić, Radenković et Likov sp. nov., *M. magnus* Vujić, Kočiš Tubić et Ačanski sp. nov., *M. nigroscutum* Vujić, Radenković et Likov sp. nov. and *M. pseudomoenium* Vujić, Kočiš Tubić et Ačanski sp. nov. An integrative taxonomy approach was used to delimit species boundaries. Two molecular markers (the mitochondrial COI gene and nuclear 28S rRNA gene—newly analysed marker for the complex) and geometric morphometry of the wing shape, together with morphological data and distribution, successfully separated all species from the complex. The morphological variability of the analysed species is described and discussed and an illustrated diagnostic key for typical morpho-forms of species from the *M. avidus* complex is presented. A distribution map of all investigated species from the complex is provided. The level of endemicity of the *M. avidus* complex was discussed.

## 1. Introduction

The hoverfly genus *Merodon* Meigen, 1803 (Diptera: Syrphidae: Eristalinae: Merodontini), with phytophagous larvae, comprises 235 described species distributed over the Palaearctic and Ethiopian regions [1,2] with some species having been introduced elsewhere, such as *Merodon equestris* into the Nearctic and New Zealand [3]. The highest species diversity has been recorded for the Mediterranean area, Middle East and Central Asia [4,5], which is connected with a high diversity of bulb species in these regions that serve as larval host plants [6]. The Iberian Peninsula and Asia Minor (Anatolian Peninsula) are considered as the largest centres of diversity and endemism of the genus, as presented by many studies of the *Merodon* fauna [2,5,7,8,9,10,11,12,13,14,15,16,17,18,19,20,21,22,23,24,25]. The high diversity of the genus *Merodon* in the Middle and Near East, the Irano-Anatolian mountains, and in Central Asia has been proven by numerous publications [10,12,14,25].

The genus *Merodon* contains five monophyletic lineages (namely *albifrons*, *aureus*, *avidus-nigritarsis*, *desuturinus* and *natans*), 24 species groups, two species subgroups and 10 unplaced species [1]. Three lineages (*aureus*, *desuturinus* and *natans*) are present in the Afrotropical Region and the Palaearctic. All groups and species from *albifrons* and *avidus-nigritarsis* lineages belong to the Palaearctic fauna. The *avidus-nigritarsis* lineage is divided into 10 species groups (*aberrans*, *aurifer*, *avidus*, *clavipes*, *fulcratus*, *italicus*, *nigritarsis*, *pruni*, *serrulatus* and *tarsatus*) and eight unplaced taxa, all together totalling 77 described species [1].

The *Merodon avidus-nigritarsis* lineage includes medium to large-sized species (11–20 mm), usually with white pollinose vittae on scutum and white pollinose fasciate maculae on terga, anterior anepisternum bare below the postpronotum, abdomen elongate, usually narrow and tapering, longer than scutum and scutellum together; posterior part of mesocoxa usually without long pile; basoflagellomere usually at most twice as long as wide; male genitalia with well-developed anterior surstylar lobe, posterior surstylar lobe and the interior accessory lobe of posterior surstylar lobe, and posterior end of lateral sclerite of the aedeagus tapering [1].

The *Merodon avidus* group covers species with elongated and tapering abdomen, at least tergum 2 with reddish-yellow lateral maculae, and reddish-yellow tarsi, including the *M. avidus* complex and species *M. femoratus* Sack, 1913, and *M. rutitarsis* Likov, Vujić et Radenković, 2016. The *Merodon avidus* species group is defined by molecular and morphological data in [25]. This species group is distributed across Europe, mainly in central and southern areas, and is less diverse in the Near and Middle East and North Africa (Algeria and Libya) [1].

The *Merodon avidus* species complex was revised in several publications [18,22,26,27,28], with four species recognised until now: *M. avidus*, *M. ibericus* Vujić, 2015, *M. megavidus* Vujić et Radenković, 2016, and *M. moenium* Wiedemann in Meigen, 1822. The *M. avidus* complex is characterised by considerable morphological variability, especially in the colouration of the antennae, thorax, abdomen and legs [28]. This colour variability has been explained by the differing availability of trophic resources during the larval stage [29]. Popović et al. [28] provided justifications for the identification of three species from the *M. avidus* complex based on new diagnostic morphological characters, records of the seasonal activity and geographical distribution of bivoltine adults, nuclear allozyme and mtDNA COI sequence analyses and descriptions of the ecological preferences of the taxa. However, some difficulties in distinguishing the species of this complex based on morphological characters remain, despite the numerous studies on the subject. Spring generations of *M. avidus* are very similar to those of *M. moenium* based on external morphology and are easily confused using existing diagnostic features (e.g., [26]). Furthermore, it is important to note that species of the *M. avidus* complex are not distinguishable by traditional visual identification of the structures of male genitalia under a stereo microscope. Ačanski et al. [22], besides traditional morphological characters, used 5′-end of the mtDNA COI gene and two different geometric morphometric approaches to quantify wing and surstylus shape variability among all hitherto-described species of the *M. avidus* complex, including the newly recognised fourth taxon *M. megavidus*. In this paper, the known and newly established morphological characters that enable us to distinguish species belonging to the *M. avidus* complex are presented.

Nowadays, with the advent of molecular and morphometric techniques, an integrative taxonomic framework has become the standard to study the taxonomy of the genus *Merodon*. Combining molecular characters (mtDNA cytochrome c oxidase subunit I (COI) and the nuclear 28S rRNA genes) with morphological traits (geometric wing morphometry, surstylus shape and size and other morphological characters), a number of cryptic and sibling species have been delineated within different species groups. Notable examples are the *ruficornis* species group [4,17], *desuturinus* species group [30], *aureus* species group [31,32,33], *nigritarsis* species group [25], *nanus* species group [34], *planifacies* subgroup [35,36], *serrulatus* species group [14], *constans* species group [13], *rufus* species group [21], *natans* species group [2], *aberrans* species group [5] and *tarsatus* species group [37].

Molecular analyses of the *Merodon avidus* species complex have been performed by studies in the last two decades [18,22,26,27,28]. Maximum parsimony analysis of the 3′-end of the COI gene revealed extensive haplotype variation in the *M. avidus* complex, distinguishing *M. bicolor* Gil Collado, 1930, (now *M. ibericus*) from the Iberian Peninsula but failing to discriminate the evolutionarily independent genetic units previously identified as *M. avidus* A (now *M. avidus*) and *M. avidus* B (now *M. moenium*) [27]. The use of the DNA barcode and construction of the NJ, ML and MP trees for identifying the species of the *M. avidus* complex led to confirmation of the presence of two clades within the complex, supporting the previous conclusion that *M. ibericus* is a separate species in the *M. avidus* complex, but did not successfully delimit sibling species *M. avidus* and *M. moenium* [28]. Further, based on the same mtDNA COI gene fragment, Ačanski et al. [22] described one more species of the complex, *M. megavidus*, validating the status of a previously recognized cryptic taxon from the Lesvos island by Ståhls et al. [18]. Cluster analyses placed *M. megavidus* as an independent branch from *M. ibericus* and *M. avidus/M. moenium* clusters [22]. Herein, we used both 5′ and 3′ COI gene fragments together with sequences of the nuclear rRNA 28S gene in the analyses of the complex.

Insect wing shape is highly heritable and an important character for separating species [38]. Geometric morphometric analysis of the wing shape has been successfully used in taxonomic studies of multiple hoverfly taxa [39,40,41,42]. Several recent studies have detected and described cryptic and sibling species complexes in the genus *Merodon* (Diptera, Syrphidae). One representative of these complexes is the *Merodon avidus* complex, which contains four sibling species that have proven difficult to distinguish using traditional morphological characters. Ačanski et al. [22] used two geometric morphometric approaches and molecular characters of the 5′-end of the mtDNA COI gene to delimit sibling taxa. A study of the relationships between 21 southern European, Moroccan and Turkish populations of the *M. avidus* species complex was carried out [28]. A cluster analysis of DNA barcoding sequences clearly separated *M. ibericus*, but not *M. avidus* and *M. moenium*, despite the lack of shared haplotypes. Analysis of molecular variance (AMOVA) and pairwise Φst values together with allozyme and ecological niche analyses revealed statistically significant variation among all species in the *M. avidus* complex. Analysis of five diagnostic enzyme loci revealed the presence of genetic differentiation among the *M. avidus*/*moenium* populations investigated (Fst = 0.654), and species-specific alleles were found at the AAT locus. The presence of two separate related taxa within the *M. avidus*/*moenium* pair of species was further supported by a UPGMA tree based on Nei’s [43] genetic distances. The value of Nei’s measure of genetic identity (I = 0.520) between two large (meta) populations of *M. avidus* and *M. moenium* suggested that these taxa are sibling species. Populations from Djerdap (Serbia) confirmed the presence of temporal divergence between these species at a locality where they occur sympatrically, while spring and autumn populations from Umag (Croatia) provide an example of morphological plasticity within the species *M. avidus*. Ecological niche analysis contributed to species delimitation. A review of the available genetic and ecological data confirmed the hypothesis that the *M. avidus* species complex, besides *M. ibericus* from the Iberian Peninsula and *M. megavidus* from the island Lesvos in Greece, consists of two sibling species in the rest of Europe, *M. avidus* and *M. moenium*, and indicated their recent speciation [22,28]. Regarding distribution and ecological preferences, several studies on hoverflies used species distribution modelling to examine the effect of climate change on species distributions [44,45] or to help in resolving taxonomic questions where traditional methods have proven inconclusive [46].

Our study had four objectives: (1) to clarify further the species boundaries of all taxa within the *M. avidus* complex using integrative taxonomy (morphological characters, geometric morphometrics of wings and molecular data); (2) to introduce a new diagnostic molecular marker in the analyses of the complex; (3) to provide descriptions and diagnostic characters of the new species; (4) to present maps for all species and discuss distribution patterns inside the complex.

## 2. Material and Methods

### 2.1. Molecular Analyses

Laboratory Procedures: Total genomic DNA was isolated from the mid and hind legs using an SDS extraction protocol [47]. Two regions (the 5′-end and 3′-end) of the mitochondrial COI gene and the nuclear D2-3 expansion fragment of the 28S rRNA gene were used in the analyses. The primers used for PCR amplification were the following: LCO1490 and HCO2198 primer pair [48] for 5′-end of COI gene, C1-J-2183 and TL2-N-3014 primer pair [49] for 3′-end of COI gene and 28S-F2 and 28S-3DR primer pair [50] for D2-3 region of 28S rRNA gene. Polymerase chain reactions (PCR) were performed according to Kočiš Tubić et al. [34]. Amplification products were enzymatically purified using Exonuclease I and Shrimp Alkaline Phosphatase enzymes (ThermoScientific, Vilnius, Lithuania) according to the manufacturer’s instructions. Sequencing was performed using forward PCR primers by the Macrogen EZ-Seq service (Macrogen Europe, Amsterdam, The Netherlands).

Data Analyses: Chromatograms of all sequences produced for this study were edited for base-calling errors using BioEdit version 7.2.5. [51]. The indel-free 5′ and 3′ sequence fragments of the protein-coding COI gene were aligned manually. Considering the presence of the indels region in the D2-3 domain of the 28S rRNA gene, the alignment of this nuclear marker required a different approach, and it was performed employing the G-INS-i strategy implemented in MAFFT version 7.0. [52].

Three sequence matrices were created, the first of which contained concatenated 5′-end and 3′-end COI gene sequences, the second one was comprised of 28S rRNA gene sequences, while in the third data matrix concatenated 5′-end and 3′-end COI and 28S rRNA gene sequences were assembled. The best nucleotide substitution model for each partition was estimated in MEGA X [53] and selected using the Akaike Information Criterion [54].

Bayesian (BI), Maximum Likelihood (ML) and Maximum Parsimony (MP) analyses were performed on assembled COI and 28S rRNA gene sequences. The sequence matrix created of concatenated 5′-end and 3′-end fragments of the COI gene was analysed using BI and ML approaches. In addition, the 28S rRNA gene sequences were clustered using the MP methodology to observe the number of mutation steps between specific taxa occurring within the analysed species group. Bayesian analyses were carried out in MrBayes 3.2.7a [55] through the CIPRES Science Gateway web portal [56]. Two independent runs of four Markov chain Monte Carlo (MCMC) permutations were performed for 10,000,000 generations, with sampling every 100 generations. Tracer 1.7.1 [57] was used to check convergence and acceptable mixing. The first 25% of the sampled iterations were discarded as burn-in, and 50% of consensus trees were computed using FigTree 1.4.4 [58]. Maximum Likelihood trees were constructed in RAxML 8.2.12 [59] using the CIPRES Science Gateway web portal [56] under the general time-reversible (GTR) evolutionary model with a gamma distribution (GTRGAMMA) [60]. Branch support was estimated with 1000 rapid bootstrap replicates. Parsimony analyses were run by NONA [61] spawned with the aid of Winclada ASADO [62], using the heuristic search algorithm with 1000 random addition replicates, holding 100 trees per round, maxtrees set to 100,000 and applying tree-bisection-reconnection branch swapping. Nodal support was assessed using non-parametric bootstrapping with 1000 replicates. As outgroups, we used *Eumerus amoenus* Loew, 1848, and species representing the main *Merodon* lineages following Vujić et al. [1]. Uncorrected sequence distance values (p-distances) among species were calculated in MEGA X [53] for concatenated 3′-end and 5′-end COI gene sequences. A list of all molecularly analysed samples with GenBank accession numbers of sequences is provided in Supplementary Information (Table S1).

### 2.2. Morphological Study

In total, 1220 specimens belonging to the *Merodon avidus* complex were studied: 792 *M. avidus*, 206 *M. moenium*, 64 *M. ibericus*, 44 *M. megavidus*, 44 *M. pseudomoenium* Vujić, Kočiš Tubić et Ačanski sp. nov., 41 *M. atroavidus* Vujić, Radenković et Likov sp nov., 23 *M. nigroscutum* Vujić, Radenković et Likov sp. nov. and 6 *M. magnus* Vujić, Kočiš Tubić et Ačanski sp nov. The examined material belongs to the following institutions and private collections: Barendregt Aat collection, the Netherlands (BA coll.); Brugge Ben collection, the Netherlands (BB coll.); Bartak Miroslav Collection, Czech Republic (BM coll.); Claussen Claus collection, Germany (CC coll.); de Courcy Williams Michael collection, Greece (CWM coll.); Doczkal Dieter collection, Germany (DD coll.); Department of Biology and Ecology, Faculty of Sciences, University of Novi Sad, Novi Sad, Serbia (FSUNS); Entomological Museum of Isparta, Isparta, Turkey (EMIT); Gilasian Ebrahim collection, Tehran, Iran (GE coll.); Hadrava Jiří collection, Czech Republic (HJ coll.); Hungarian Natural History Museum, Budapest, Hungary (HNHM); Krpač Vladimir collection, North Macedonia (KV coll.); The Melissotheque of the Aegean, University of the Aegean, Mytilene, Greece (MAegean); Musée National d’Histoire Naturelle, Paris, France (MNHN); Finnish Museum of Natural History, University of Helsinki, Helsinki, Finland (MZH); Naturhistorisches Museum Wien, Vienna, Austria (NHMW); National Museum Prague, Prague, Czech Republic (NMPC); Palmer J. Chris collection, UK (PJC coll.); Naturalis Biodiversity Center, Leiden, the Netherlands (RMNH); Ssymank Axel collection, Germany (SA coll.); Speight C.D. Martin collection, Ireland (SCDM coll.); Schmalhausen Institute of Zoology, National Academy of Sciences, Kiev, Ukraine (SIZK-I.I.); van Steenis Jeroen collection, the Netherlands (SJ coll.); Stuke Jens-Hermann collection, Germany (SJH coll.); Smit T. John collection, the Netherlands (STJ coll.); Van de Weyer Guy collection, Belgium (VWG coll.); World Museum Liverpool, Liverpool, UK (WML); Zoologisches Museum of the Humboldt University, Berlin, Germany (ZHMB) Zoologisches Forschungsinstitut und Museum Alexander Koenig, Bonn, Germany (ZFMK).

The examined individuals were collected by multiple researchers over a hundred years (1901–2020) across a vast study area covering most of Europe, North Africa and the Middle East. The distribution maps were generated using QGIS ver. 3.2.22—Białowieża [63].

To study the male genitalia, dry specimens were relaxed in a humidity chamber, after which the genitalia were separated from the rest of the specimen using an entomological pin. In the next step, the genitalia were cleared by boiling them individually in a 10% KOH solution for a few minutes and dipping them in acetic acid and ethanol. Genitalia were stored in microvials containing glycerol.

**Figure 1:**
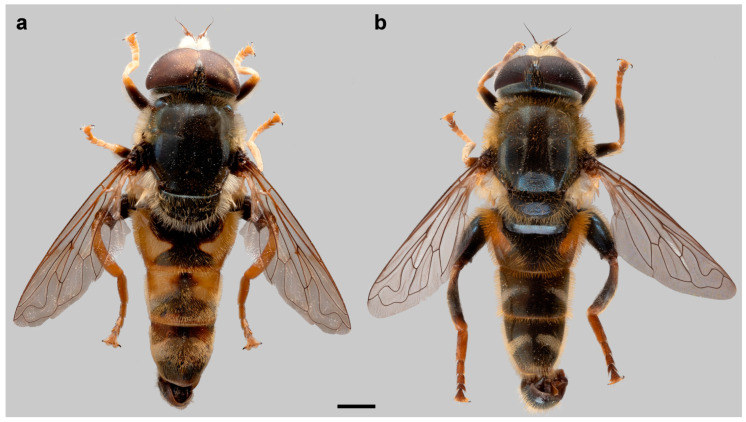
Habitus of male (**a**) *Merodon avidus* and (**b**) *M. moenium*, dorsal view. Scale bar 2 mm.

For drawing, a Leica MZ16 binocular microscope with an FSA 25 PE drawing tube was used, while photographs were made using Nikon Coolpix D7100 digital camera attached to a Nikon SMZ 745T stereomicroscope. After that, the photographs were stacked in CombineZ software, version 5 [64]. Figure 1 was taken with Canon EOS 6D Mark II digital camera connected to a Canon MP-E 65MM lens and processed in Helicon image stacking software 8.2.2. Measurements were taken with an eyepiece graticule or micrometre.

The terminology used in the morphological descriptions follows Thompson [65]. The terms of the male genitalia follow Marcos-García et al. [7], while “fasciate maculae” follow Vujić et al. [1].

### 2.3. Type Material

For holotypes and paratypes, the original label data have been given verbatim (with added English translations of Serbian phrases); quotation marks (“ ”) were used to indicate separate labels; a slash (/) has been used to indicate separate lines within a label.

### 2.4. Geometric Morphometric Analysis

Wing shape variation was observed in 472 specimens of *M. avidus* (181), *M. moenium* (175), *M. ibericus* (32), *M. atroavidus* sp. nov. (19), *M. magnus* sp. nov. (6), *M. megavidus* (23), *M. nigroscutum* sp. nov. (17) and *M. pseudomoenium* sp. nov. (19) by using the landmark-based, geometric morphometric method [66]. Variations in wing shape were assessed among the species and the geographically defined groups of specimens (herein treated as populations).

**Figure 2:**
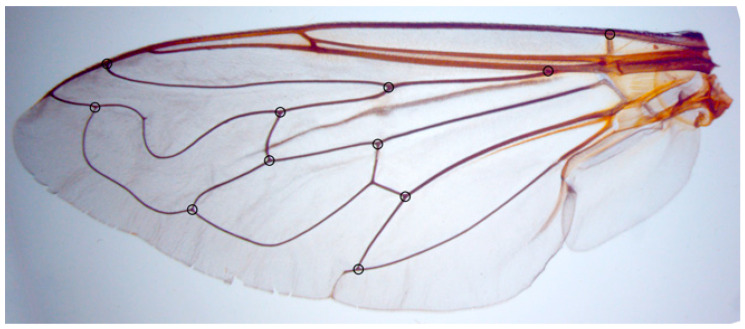
*Merodon moenium*, the location of 11 landmarks on a left wing selected for geometric morphometric analysis.

The left wing of each specimen was taken off using micro-scissors and mounted in Hoyer’s medium on a microscopic slide. Wings were archived and labelled with a unique code in the FSUNS and other data relevant to the specimens. Eleven homologous landmarks that could be reliably identified at vein intersections or terminations were selected using TpsDig 2.05 [67] (Figure 2). Generalised least squares Procrustes superimposition on the raw coordinates was carried out using MorphoJ ver. 2.0 [68] to minimise non-shape variations in location, scale and orientation of wings and to superimpose the wings in a common coordinate system [69,70]. Principal component analysis was carried out on the Procrustes shape variables to reduce the dimensionality of the data set. All further statistical analyses were conducted in the reduced space using a subset of independent principal components (PCs) that describe the highest overall classification percentage calculated in stepwise discriminant analysis [71]. Also, to analyse sexual dimorphism, two-way MANOVA (sex, species) was used. Besides PCA, which depict the position of individuals in PCA defined space, discriminant function (DA) and canonical variate (CVA) analyses were employed to explore wing shape variation among species groups. DA with cross-validation was produced in MorphoJ ver. 2.0 [68]. Additionally, a Gaussian naïve Bayes classifier was also used to delimit species boundaries based on wing shape variation without a priori-defined group. Phenetic relationships among the species and populations were characterised using an unweighted pair group method with arithmetic mean cluster analysis (UPGMA) based on squared Mahalanobis distances computed from the discriminant function analysis. Stepwise discriminant analysis, PCA, MANOVA, CVA, DA, Gaussian naïve Bayes classifier and UPGMA were performed in Statistica for Windows version 13 [72].

## 3. Results

### 3.1. Molecular Analyses

In total, 63 *Merodon avidus* species complex specimens were molecularly analysed. Amplification of both the 5′-end and 3′-end of the COI gene was successful for all individuals, and sequences were combined in one dataset with the final length of 1346 nucleotides, among which the 5′ fragment contained 620 aligned characters and the 3′ fragment had a final length of 726 nucleotides. On the other hand, 28S rRNA amplicons were obtained for 48 specimens, and the alignment of this nuclear region contained 544 characters. After including outgroups into the dataset, indel regions caused the extension of the 28S rRNA sequences alignment total length to 566 characters.

**Figure 3:**
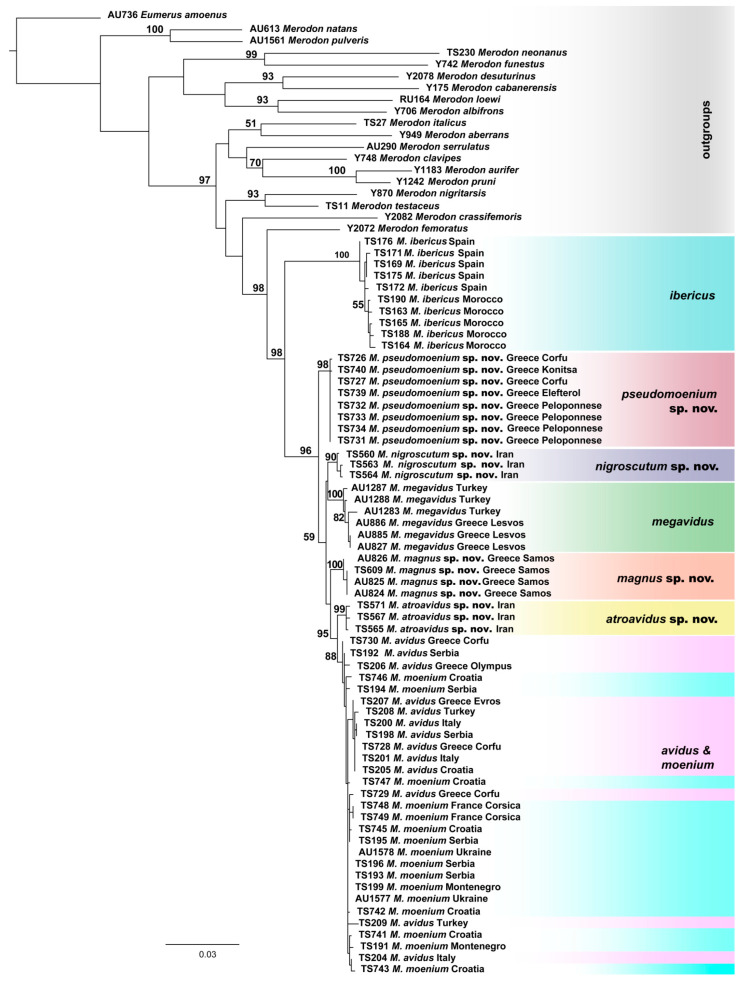
Maximum Likelihood tree based on the concatenated COI gene fragments (5′-end and 3′-end) (bootstrap support values (≥50) are shown near nodes).

The taxonomic boundaries of the newly described species (*M. atroavidus* sp. nov., *M. magnus* sp. nov., *M. nigroscutum* sp. nov. and *M. pseudomoenium* sp. nov.) were detected with results obtained from the cluster analyses based on the concatenated COI gene fragments (5′-end and 3′-end) (Figure 3 and Figure S1). However, COI sequences failed to distinguish *M. avidus* and *M. moenium* species. Conversely, the distinction between these two species was supported by the DNA polymorphism within the 28S rRNA gene. At the same time, the same nuclear marker has not been proven useful in identifying all newly described species (Figure S2).

**Figure 4:**
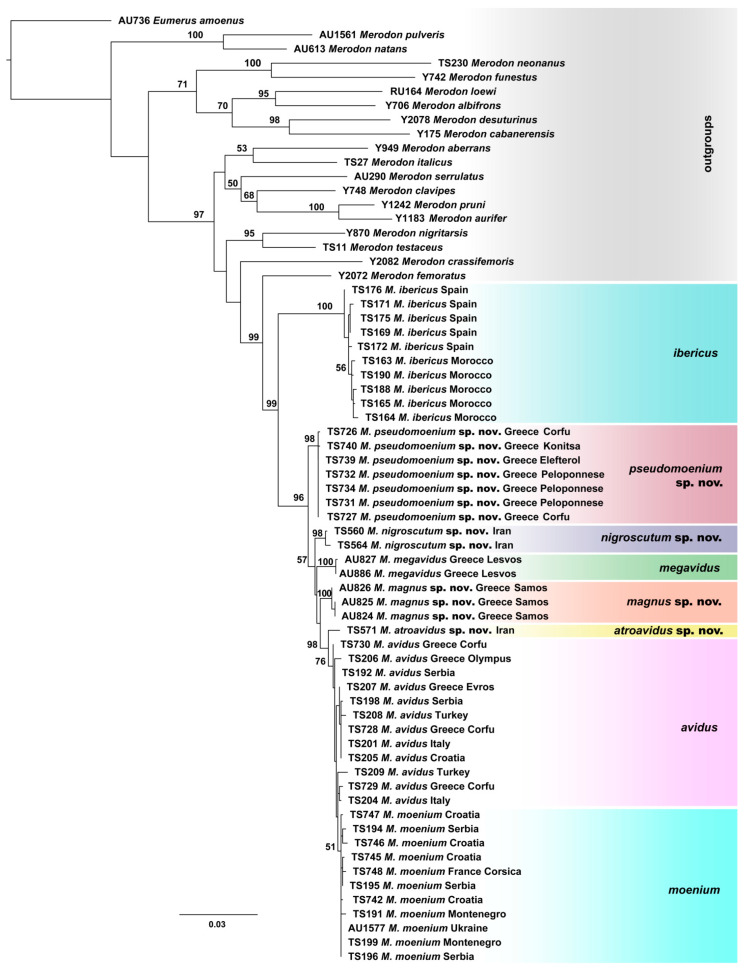
Maximum Likelihood tree based on combined COI gene fragments (5′-end and 3′-end) and 28S rRNA gene sequences (bootstrap support values (≥50) are shown near nodes).

All conducted phylogenetic analyses (Maximum Likelihood, Bayesian and Maximum Parsimony) were performed on the dataset which included combined 5′-end and 3′-end COI and 28S rRNA genes sequences, resulted in a delimitation of all species within the *M. avidus* complex (Figure 4 and Figures S3 and S4). The same tree topology has been obtained for all employed methods, revealing a clear distinction of the newly described species and a successful separation between *M. avidus* and *M. moenium*.

Calculated p-distances for the concatenated 3′ and 5′ COI gene fragments ranged from 0.39% to 4.77% among species from the *M. avidus* complex (Table S2). Specifically, the uncorrected pairwise distances that distinguish four new species within the *M. avidus* complex were in the range from 1.38–4.55% (for *M. nigroscutum* sp. nov.), 0.95–4.77% (for *M. atroavidus* sp. nov.), 1.33–4.35 (for *M. magnus* sp. nov.) and 1.33–4.37% (for *M*. *pseudomoenium* sp. nov.).

### 3.2. Geometric Morphometric Analysis

#### 3.2.1. Sexual Dimorphism

The two-way MANOVA of wing shape showed highly significant differences among species (F_126, 3333_ = 9.16013; *p* < 0.01), sexes (F_18, 506_ = 24.6197; *p* < 0.01) and their interaction (F_126, 3333_= 1.57895; *p* < 0.01), indicating that there is sexual shape dimorphism. Due to sexual dimorphism, male and female specimens were analysed separately.

#### 3.2.2. Males

Principal component analysis carried out on the Procrustes shape variables produced 18 PCs (Table S3). Position of specimens in PCA space is depicted on Figure S5. Stepwise discriminant analysis revealed that all PCs represented the highest overall classification percentage of investigated taxa. Discriminant analysis showed that males of all species pairs differed highly significantly in wing shape (Table S4). Notably, 92.5% of specimens were correctly classified into a priori defined groups. All male specimens of *M. megavidus* and *M. pseudomoenium* sp. nov. were correctly classified, whereas *M. atroavidus* sp. nov. and *M. nigroscutum* sp. nov. had the lowest classification success, at 62.5% and 40%, respectively. However, all *M. nigroscutum* sp. nov. specimens were correctly classified by the Gaussian naïve Bayes classifier (100%). Additionally, *M. atroavidus* sp. nov. had the highest classification success using the Gaussian naïve Bayes classifier (75%). The cross-validation test indicated a relatively high percentage of correct classification in most species, except for *M. nigroscutum* sp. nov., *M. atroavidus* sp. nov. and *M. pseudomoenium* sp. nov. (Table S5). These species exhibited the lowest classification accuracy, particularly noticeable in comparisons involving species represented by a limited number of individuals.

**Figure 5:**
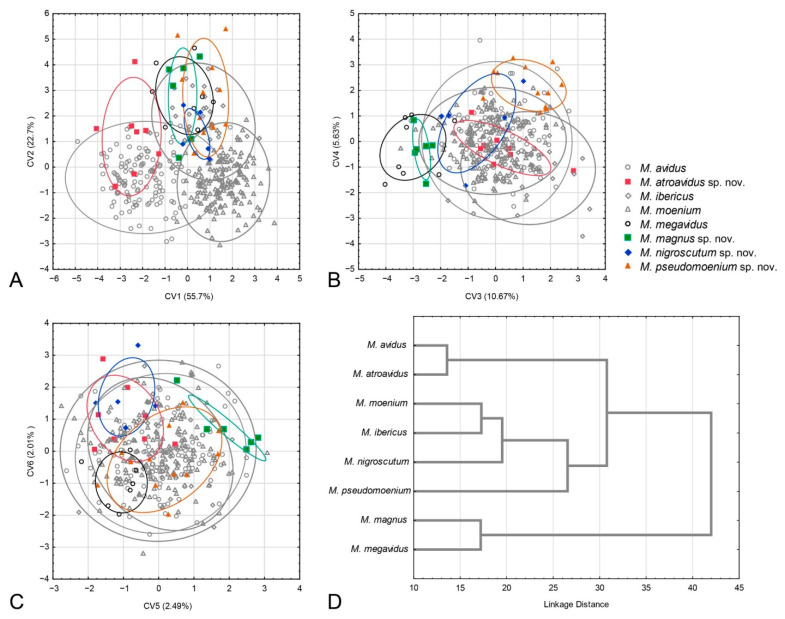
Geometric morphometric analysis of the wing shape in males. (**A**) Position of male specimens in the space defined by CV1 and CV2 axes, (**B**) position of male specimens in the space defined by CV3 and CV4 axes, (**C**) position of male specimens in the space defined by CV5 and CV6 axes, (**D**) UPGMA phenogram constructed using squared Mahalanobis distances of wing shape.

**Figure 6:**
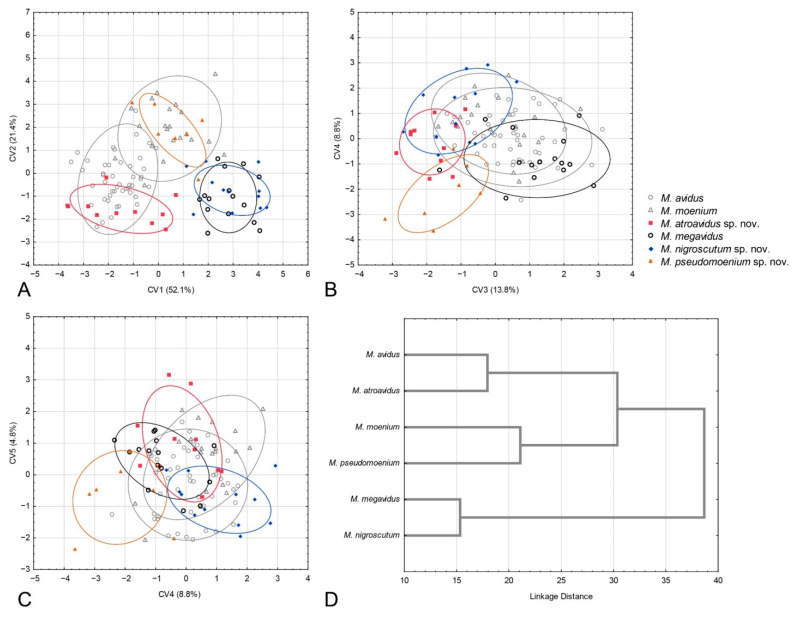
Geometric morphometric analysis of the wing shape in females. (**A**) Position of female specimens in the space defined by CV1 and CV2 axes, (**B**) position of female specimens in the space defined by CV3 and CV4 axes, (**C**) position of female specimens in the space defined by CV4 and CV5 axes, (**D**) UPGMA phenogram constructed using squared Mahalanobis distances of wing shape.

The succeeding CVA gave six highly significant canonical axes (CV1: Wilks’ = 0.0507, χ^2^ = 1031.426, *p* < 0.01; CV2: Wilks’ = 0.1869, χ^2^ = 575.319, *p* < 0.01; CV3: Wilks’ = 0.4015, χ^2^ = 315.734, *p* < 0.01; CV4: Wilks’ = 0.6111, χ^2^ = 170.429, *p* < 0.01; CV5: Wilks’ = 0.7789, χ^2^ = 86.460, *p* < 0.01; CV6: Wilks’ = 0.8758, χ^2^ = 45.866, *p* < 0.01). The first canonical axis represents the majority of wing shape variation (55.7%) and differentiates *M. atroavidus* sp. nov. from *M. moenium*, *M. megavidus*, *M. magnus* sp. nov., *M. pseudomoenium* sp. nov. and *M. ibericus* (Figure 5A). This axis also indicates wing shape differentiation between *M. avidus* and *M. moenium*. The second CV, with 22.7% of total wing shape variation, separates *M. moenium* from *M. pseudomoenium* sp. nov., *M. magnus* sp. nov., *M. megavidus* and *M. ibericus* (Figure 5A). CV3, with 10.67% of total wing shape variation, separates *M. magnus* sp. nov. and *M. megavidus* from *M. atroavidus* sp. nov., *M. pseudomoenium* sp. nov., *M. ibericus* and *M. nigroscutum* sp. nov., whereas CV4, with 5.63%, clearly separates *M. pseudomoenium* sp. nov. from *M. megavidus*, *M. magnus* sp. nov., *M. atroavidus* sp. nov. and *M. ibericus* (Figure 6B). Clear separation of *M. magnus* sp. nov. from *M. nigroscutum* sp. nov., *M. atroavidus* sp. nov. and *M. megavidus* is depicted on CV5 (2.49%), while CV6, with 2.01% of total wing shape variation, clearly separates *M. nigroscutum* sp. nov. from *M. megavidus* (Figure 5C). The dendrogram derived by UPGMA clustering of squared Mahalanobis distances shows the phenetic relationships among the males of the analysed species according to wing shape (Figure 5D).

#### 3.2.3. Females

A PCA was conducted on the Procrustes shape variables, yielding 18 PCs, as detailed in Table S3. The spatial distribution of specimens within the PCA framework is illustrated in Figure S5. Subsequent stepwise discriminant analysis showed that these PCs achieved the highest overall accuracy in classifying the taxa under investigation. Also, the discriminant analysis provided evidence for highly significant wing shape differences among females of all species pairs (Table S6). Females of *M. ibericus* and *M. magnus* were not included in the analysis due to insufficient specimens. Additionally, DA showed correct species assignment for 89.29% of female specimens. All specimens of *M. nigroscutum* sp. nov. and *M. pseudomenium* sp. nov. were correctly classified. *Merodon avidus*, *M. atroavidus* sp. nov., *M. megavidus* and *M. moenium* were correctly classified for 93.78%, 81.82%, 80% and 80.95% of specimens, respectively. A congruent classification success was obtained using Gaussian naïve Bayes classifier (*Merodon avidus* 93.48%, *M. atroavidus* sp. nov. 81.82%, *M. megavidus* 93.33%, *M. moenium* 80.95%*, M. nigroscutum* sp. nov. 100% and *M. pseudomenium* sp. nov. 100%). The cross-validation test showed a high success rate in accurately classifying most species pairs. However, *M. nigroscutum* sp. nov., *M. atroavidus* sp. nov. and *M. pseudomoenium* sp. nov. were exceptions, as detailed in Table S5. These particular species had lower classification accuracy, and as in males, this trend is most apparent in cases where species with fewer individual representations were compared.

The CVA conducted on wing shape parameters gave four highly significant and one significant canonical axis (CV1: Wilks’ = 0.0212, χ^2^ = 381.323, *p* < 0.01; CV2: Wilks’ = 0.0964, χ^2^ = 231.551, *p* < 0.01; CV3: Wilks’ = 0.239, χ^2^ = 141.682, *p* < 0.01; CV4: Wilks’ = 0.4669, χ^2^ = 75.392, *p* < 0.01; CV5: Wilks’ = 0.7508, χ^2^ = 23.369, *p* < 0.05). CV1, with 52.1% of total wing shape variation, clearly separates *M. avidus* and *M. atroavidus* sp. nov. from *M. megavidus* and *M. nigrosuctum* sp. nov. (Figure 6A). It also indicates the separation of *M. pseudomoenium* sp. nov. from the four species mentioned above (Figure 6A). The second canonical axis, with 21.4% of total shape variation, clearly separates *M. pseudomoenium* sp. nov. from *M. atroavidus* sp. nov., *M. megavidus* and *M. nigroscutum* sp. nov. (Figure 6A). CV3, with 13.8% wing shape variation, clearly differentiates *M. atroavidus* sp. nov. from *M. megavidus* and indicates separation of *M. atroavidus* sp. nov., *M pseudomoenium* sp. nov. and *M. nigroscutum* sp. nov. from *M. megavidus*, *M. avidus* and *M. moenium* (Figure 6B). *Merodon nigroscutum* sp. nov. and *M. pseudomoenium* sp. nov. are clearly separated by CV4 (8.8%), and this axis also indicates separation of *M. pseudomoenium* sp. nov. from all other species (Figure 6B). CV5, with smaller shape variation, depicts wing shape differences between *M. pseudomoenium* sp. nov. and *M. nigroscutum* sp. nov., and *M. atroavidus* sp. nov., *M. moenium* and *M. megavidus* (Figure 6C). Phenetic relationship among females based on wing shape is depicted at Figure 6D.

#### 3.2.4. Population Analysis

**Figure 7:**
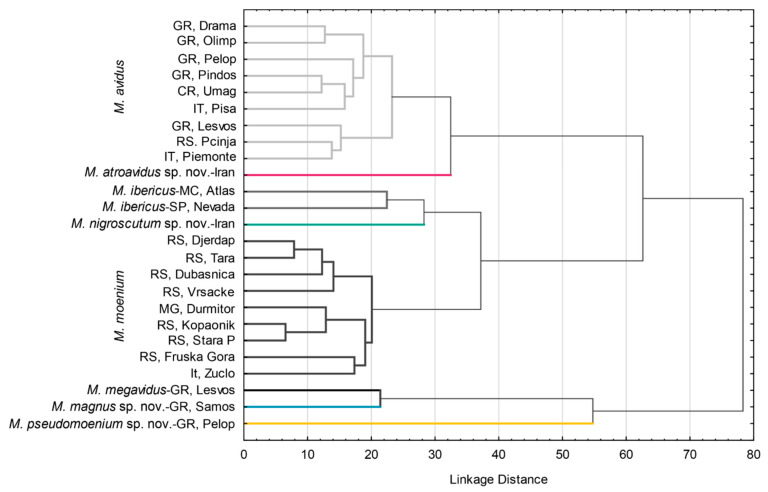
UPGMA phenogram constructed using squared Mahalanobis distances of wing shape for populations of species of the *Merodon avidus* complex.

Phenetic relations among populations were accessed using DA and depicted using UPGMA cluster analysis constructed with the Mahalanobis square distances (Figure 7). Three main clusters were formed. Within all clusters, conspecific populations were grouped (Figure 7). The first cluster consists of *M. avidus* and *M. atroavidus* sp. nov., with a clear separation of *M. atroavidus* sp. nov. The second cluster comprises *M. ibericus*, *M. nigroscutum* sp. nov. and *M. moenium*, whereas the third cluster is made of *M. megavidus*, *M. magnus* sp. nov. and *M. pseudomoenium* sp. nov. populations (Figure 7).

### 3.3. Morphology and Taxonomy

#### 3.3.1. *Merodon avidus* Group

**Figure 8:**
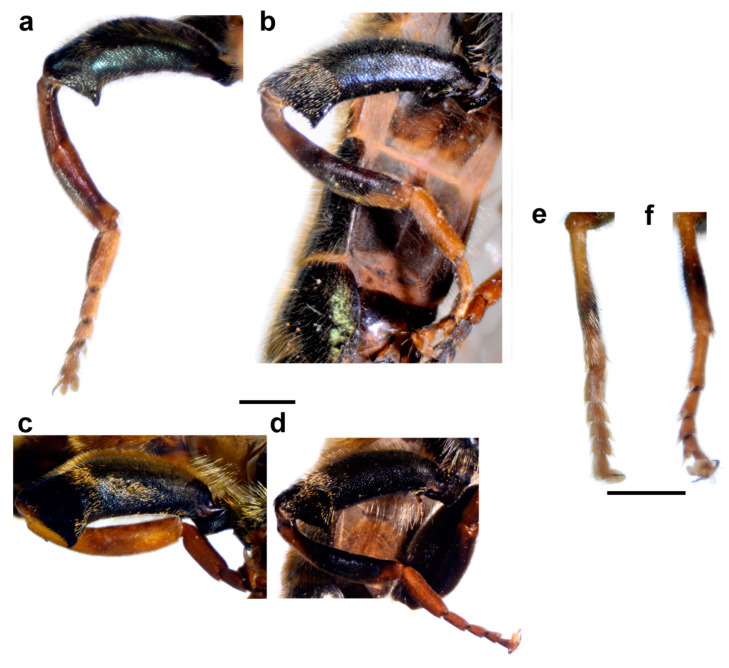
Legs of male. (**a**) *Merodon atroavidus*, (**b**) *M. nigroscutum*, (**c**) *M. magnus*, (**d**) *M. pseudomoenium* and (**e**,**f**) *M. atroavidus*. (**a**–**d**) metaleg, lateral view, (**e**) protibia and –tarsus, dorsal view and (**f**) mesotibia and –tarsus, dorsal view (scale bar (**a**–**d**) 1 mm, (**e**,**f**) 0.5 mm).

**Figure 9:**
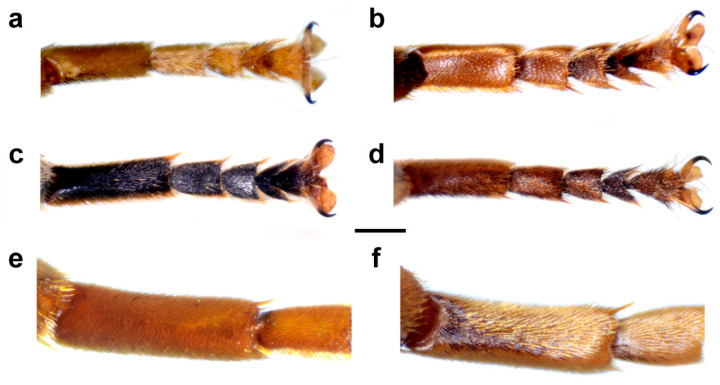
Metatarsus of male, dorsal view. (**a**) *Merodon avidus*, (**b**) *M. femoratus*, (**c**) *M. nigritarsis*, (**d**) *M. atroavidus* and (**e**,**f**) *M. megavidus* (scale bar (**a**–**d**) 0.5 mm, (**e**,**f**) 0.75 mm).

**Figure 10:**
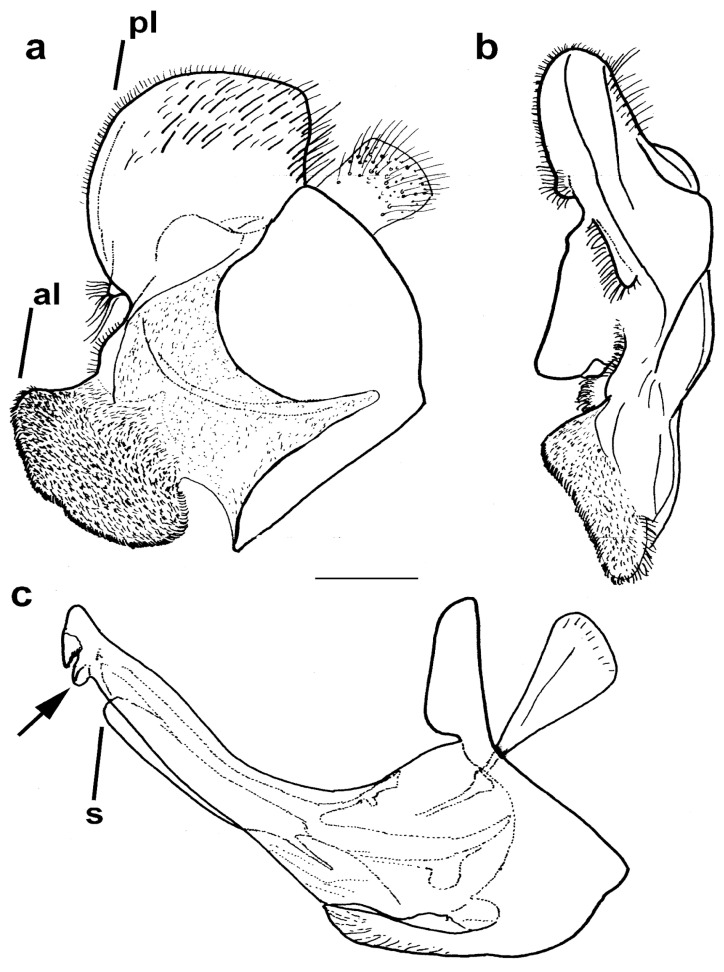
Male genitalia *Merodon avidus*. (**a**) epandrium, lateral view, (**b**) epandrium, ventral view and (**c**) hypandrium, lateral view (scale bar 0.2 mm). pl—posterior surstylar lobe; al—anterior surstylar lobe; s—lateral sclerite of aedeagus; the ctenidium marked with arrow.

**Figure 11:**
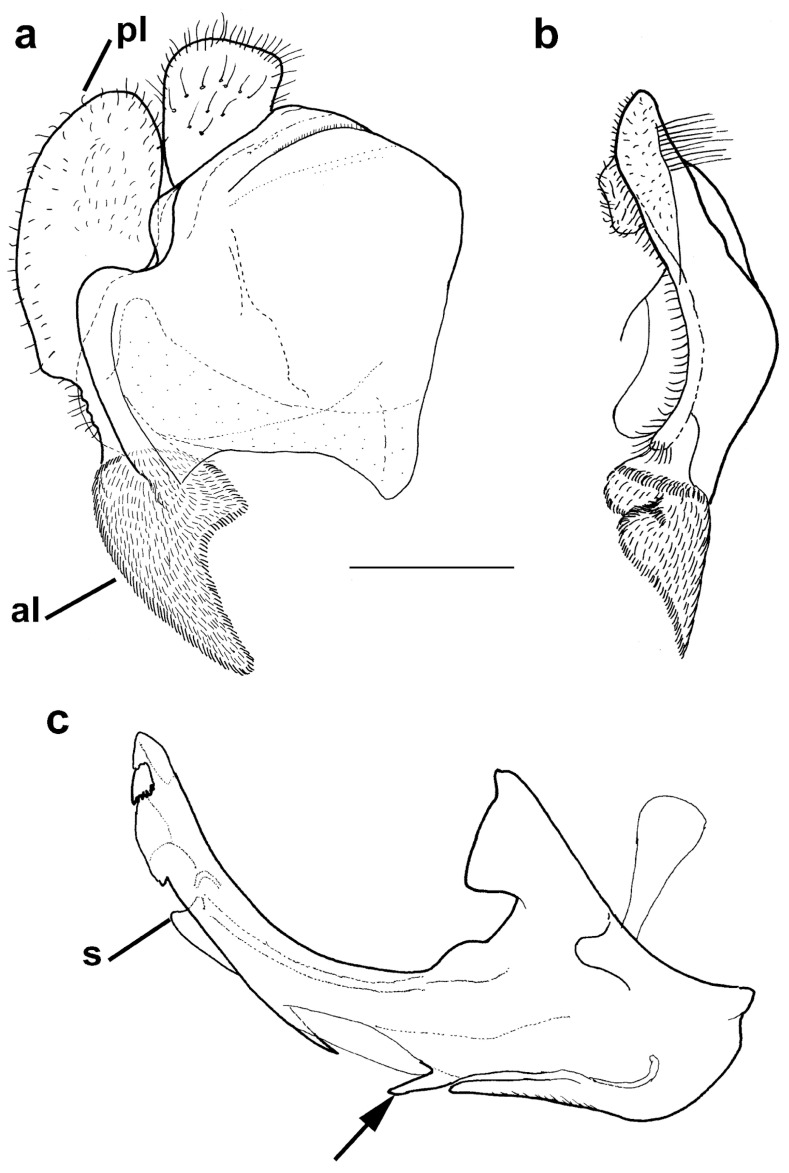
Male genitalia *Merodon nigritarsis*.(**a**) epandrium, lateral view, (**b**) epandrium, ventral view and (**c**) hypandrium, lateral view (scale bar 0.5 mm). pl—posterior surstylar lobe; al—anterior surstylar lobe; s—lateral sclerite of aedeagus; the subapical thorn marked with arrow.

Diagnosis. Medium to large-sized species (11–17 mm) (Figure 1); black mesonotum with four white pollinose longitudinal vittae on scutum; tapering orange and black abdomen with white, pollinose fasciate maculae on terga 2–4 (exceptionally absent on tergum 2); tarsi reddish-orange dorsally; metafemur moderately wide and slightly curved (as in Figure 8a–d and Figure 20), with short pile posteroventrally. Similar to *Merodon nigritarsis* group, but differs by the red-yellow tarsi in the *M. avidus* group (as in Figure 9a,d–f; except in *M. femoratus* with apical tarsomeres that can be partly brown dorsally, Figure 9b), while dark brown dorsally in the *M. nigritarsis* group (Figure 9c); by lack of subapical thorns on ventral margin of hypandrium (as in Figure 10c), while present in the *M. nigritarsis* group (Figure 11c: marked with arrow); projections just behind the ctenidium can be present (Figure 10c: marked with arrow), while they are absent in the *M. nigritarsis* group (Figure 11c); and by the differences in the shape of the lateral sclerit of aedeagus (Figure 10c and Figure 11c: s) and surstylus (Figure 10a and Figure 11a: al, pl).

The *Merodon avidus* group includes *M. avidus* complex, *M. femoratus* Sack, 1913, and *M. rutitarsis*. This species group is distributed all across Europe, mainly in central and southern zones, in the Near and Middle East and in North Africa (Morocco, Algeria and Libya).

#### 3.3.2. *Merodon avidus* Complex

*Merodon avidus* complex contains a group of taxa with extremely similar shape of male genitalia (Figure 10).

### 3.4. Description of New Species

*Merodon atroavidus* Vujić, Radenković et Likov sp nov.

urn:lsid:zoobank.org:act:1DC2D156-464A-49A4-AB10-7E86BE3EC33D

Type material. Holotype: 1 ♂, pinned. Original labels: “IRAN: Mázandarán prov./34km S of Chalus/36.4253972, 51.5101750/5. VI. 2015 1036m/P. Baňař lgt.”, “Potential holotype has to/be deposited in Moravian/Museum in Brno,/Czech Republic,/PetrBanar@seznam.cz”, “18317”, “WM 2981”. Paratypes: IRAN: 3 ♂♂; 34km S of Chalus; 36.4253972, 51.5101750; 5 June 2015; P. Banar leg; FSUNS ID 18338, 18342, 18343 19♀♀; same data as for preceding; FSUNS ID 18303, 18318, 18319, 18320, 18321, 18322, 18323, 18324, 18325, 18326, 18327, 18328, 18329, 18330, 18331, 18332, 18333, 18334, 18339 4♂♂; 3.2km S of Kandelous; 36.2981528, 51.5774917; 4 June 2015; P. Banar leg.; FSUNS ID 18340, 18341, 18344, 18345 3♀♀; same data as for preceding; FSUNS ID 18335, 18336, 18337; AZERBAIJAN: 5 ♂♂; Altyagach; 40.833, 48.83; 23 June 1996; M. Hauser leg.; ZFMK (ZFMK-DIP-00069609, ZFMK-DIP-00069610, ZFMK-DIP-00069611, ZFMK-DIP-00069613, ZFMK-DIP-00069618); FSUNS ID 25333, 25334, 25335, 25336, 25337; all det. M. Hauser as *Merodon avidus* 1 ♂; Avash; 38.83, 48.17; 15 June 1996; M. Hauser leg.; ZFMK (ZFMK-DIP-00069614); FSUNS ID 25339; det. M. Hauser as *Merodon avidus*; 1 ♂; Avash; 38.83, 48.17; 16 June 1996; M. Hauser leg.; ZFMK (ZFMK-DIP-00069612); FSUNS ID 25338; det. M. Hauser as *Merodon avidus* 3 ♂♂; Apo below Bilasar; 38.58, 48.759; June 1996; M. Hauser leg.; ZFMK (ZFMK-DIP-00069615, ZFMK-DIP-00069616, ZFMK-DIP-00069619); FSUNS ID 25341, 25342, 25343; all det. M. Hauser as *Merodon avidus* 1 ♂; Istisu W Astara; 38.33, 48.75; 4 June 1996; M. Hauser leg.; ZFMK (ZFMK-DIP-00069617); FSUNS ID 25344; det. M. Hauser as *Merodon avidus*.

**Figure 12:**
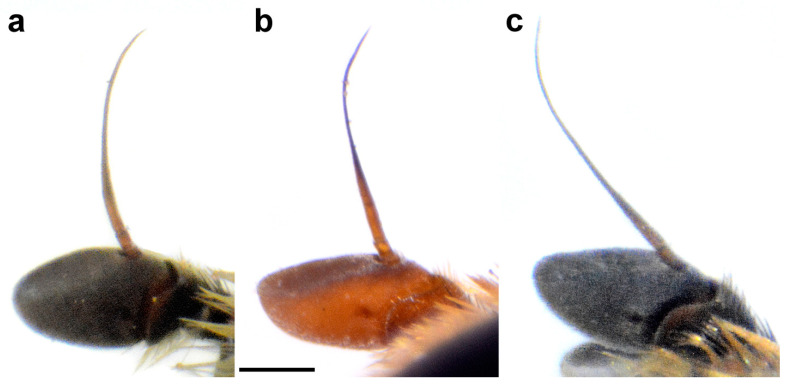
Basoflagellomere of male, lateral view. (**a**) *M. atroavidus*, (**b**) *M. magnus* and (**c**) *M. nigroscutum* (scale bar 0.5 mm).

Diagnosis. Large to medium sized species (13–15 mm). Terga partly reddish, at least laterally, in female terga 2 and 3 with reddish lateral triangular maculae (Figure 14a and Figure 15a). Scutum with black pile usually at wing bases (Figure 16a: marked with red arrow). Legs: metatibia reddish brown (Figure 8a), protibia and mesotibia with brown medial ring (Figure 8e,f); tarsi in some specimens dorsally partly brown (Figure 9d). Antenna black to dark brown (Figure 12a). Surstylus in Figure 17a. Similar to *M. avidus*, from which it clearly differs in black antenna, wing morphometry, molecular data and distribution (Figure 3, Figure 4, Figure 5, Figure 6, Figure 18 and Figures S1, S3 and S4).

Description. Male.

**Figure 13:**
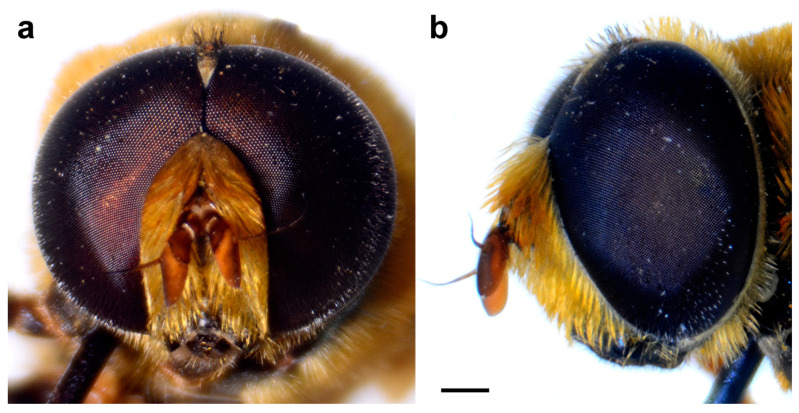
*Merodon magnus*, head of male. (**a**) frontal view, (**b**) lateral view (scale bar 0.5 mm).

Head. Antenna (Figure 12a) dark brown to black, basoflagellomere 1.8–2.0 times as long as wide, 2.0 times longer than pedicel, concave, with acute apex; arista pale, but dark brown in apical ⅔, and thickened basally, 1.4 times longer than basoflagellomere; covered with short, dense microtrichia. Face and frons black, covered with long golden-yellow pile and silver, dense pollinose. Oral margin shiny black, except for the lateral pollinose areas (as in Figure 13a). Vertical triangle isosceles, shiny black except in front of the anterior ocellus that has pale pollinosity, covered with long orange-yellow pile except for black pile on the ocellar triangle. Ocellar triangle equilateral. Eye contiguity about 12 ommatidia long. Vertical triangle:eye contiguity:ocellar triangle = 1.5:0.7:1. Eye pile dense, white. Occiput with orange to yellow pile, along the eye margin with dense white pollinosity and posteriorly with metallic, bluish-greenish lustre.

Thorax. Mesonotum black with bronze lustre, covered with relatively long, dense, erect golden pile. Scutum above the wing-base with a patch of black pile. Scutum with two lateral and two submedian, longitudinal, white pollinose vittae. Posterior anepisternum, anteroventral and posterodorsal part of katepisternum, anepimeron, metasternum and katatergum with long golden to yellow pile and grey-green pollinose. Wing hyaline, with dense microtrichia; veins dark brown except for light brown C, Sc and R1. Calypter pale yellow. Haltere with light brown pedicel and yellow capitulum. Legs orange to yellow, except for the black metafemur and basal ¾ of the pro- and mesofemora. Pile on legs yellow to golden. Metafemur (Figure 8a) medium wide and slightly curved, about 3.6 times as long as wide. Pile on metafemur short posteroventrally.

**Figure 14:**
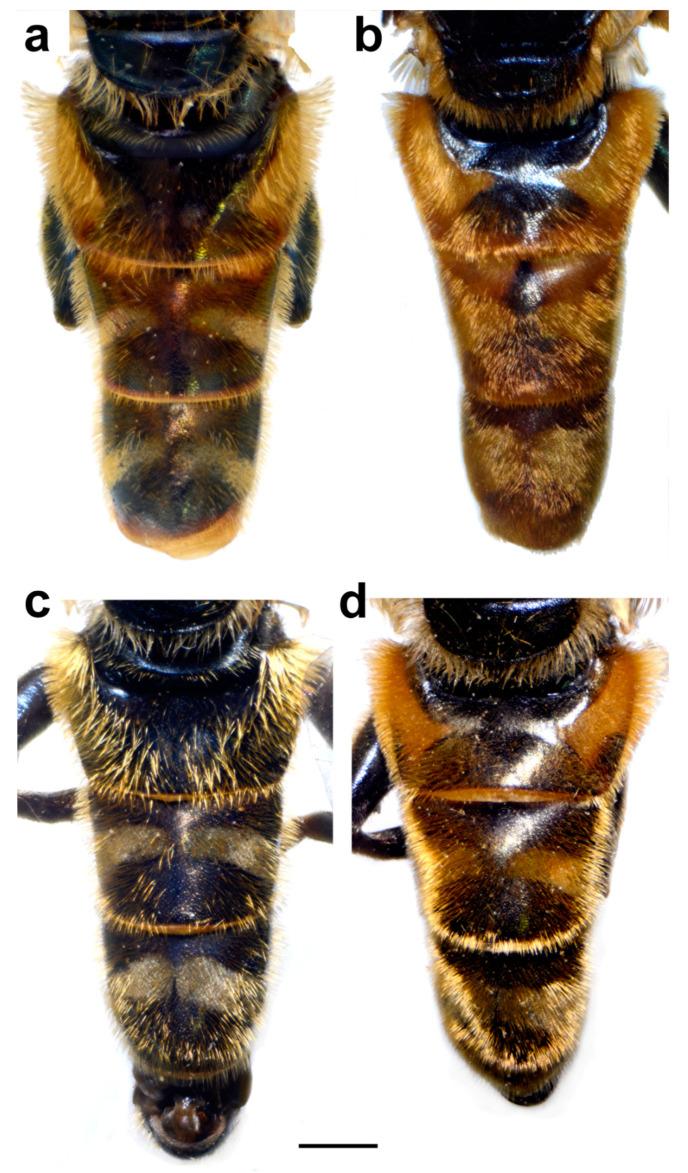
Abdomen of male, dorsal view. (**a**) *M. atroavidus*, (**b**) *M. magnus*, (**c**) *M. nigroscutum* and (**d**) *M. pseudomoenium* (scale bar 1 mm).

Abdomen. Dark, with a pair of white or grey, pollinose fasciate maculae, tapering, 1.4 times longer than mesonotum (including scutellum). Terga partly reddish at least laterally, except for black tergum 1 and central parts of terga 2–4 (Figure 14a). Terga 2–4 each with a pair of white pollinose fasciate maculae (Figure 14a). Pile on terga golden-yellow. Sterna translucent, orange to brown towards the tip of the abdomen, covered with long yellow to whitish pile.

**Figure 15:**
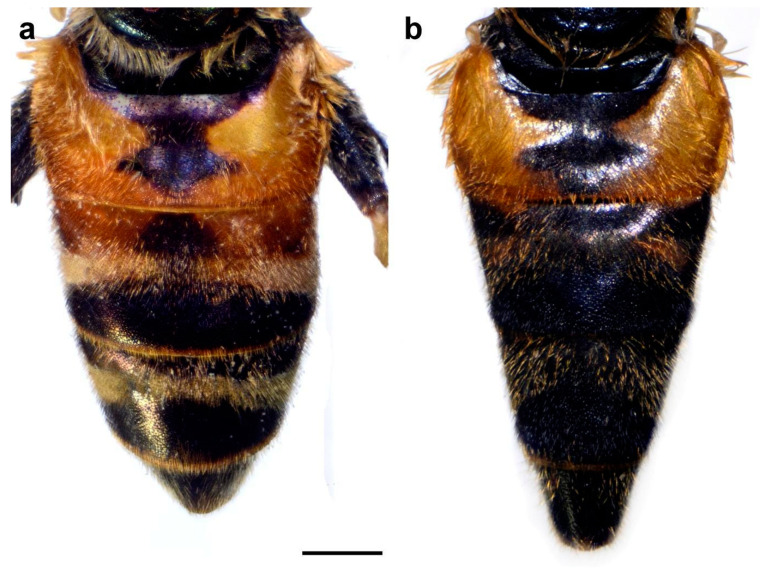
Abdomen of female, dorsal view. (**a**) *M. atroavidus*, (**b**) *M. nigroscutum* (scale bar 1 mm).

Female. Similar to the male except for typical sexual dimorphism and for the following characteristics: basoflagellomere broader and longer; frons usually with two wide (about 1/3 width of frons), lateral, silver pollinose longitudinal vittae; frons in the widest part about 0.25 width of head; white pollinose, longitudinal vittae on scutum more visible; terga 2 and 3 with reddish lateral triangular maculae (Figure 15a); white pollinose, transverse fasciate maculae present on terga 3–4 (Figure 15a); terga 2–3 with black pile on dark parts; white pollinose fasciate maculae solely with pale pile (Figure 15a).

Distribution. Caspian region of Iran and Azerbaijan (Figure 18).

Etymology. The name *atroavidus* is derived from the Latin adjective “ater”, meaning black, dark-coloured, and the name *avidus* of nearly related species. It refers to the black antenna of *Merodon atroavidus* sp. nov. compared with *M. avidus*.

*Merodon magnus* Vujić, Kočiš Tubić et Ačanski sp nov.

urn:lsid:zoobank.org:act:12165CFA-AE07-49DE-B92B-5507624E483E

Type material. Holotype: 1 ♂, pinned, with genitalia in a separate microvial with glycerine. The left wing glued to a separate label. Original labels: “Greece: Samos, Pyrgos-/ulaz u klisuru [*eng:* entry to the gorge] 37.711/26.804 08/06/2012”, “V74”, “WM2333”, “AU824”. Paratypes: GREECE: 1 ♂; Samos, Pyrgos-ulaz u klisuru; 37.709742 26.800574; 8 June 2012; A. Vujić leg.; FSUNS ID V76 1 ♀; same data as for preceding; FSUNS ID V75 2 ♂♂; Samos, Kosmadei; 37.760, 26.660; June 2010; A. Vujić leg.; FSUNS ID C17, C21 1 ♀; same data as for preceding; FSUNS ID C18.

Diagnosis. Large species (15–17 mm), with orange-yellow tibiae, tarsi and basoflagellomere, and orange and dense pilosity on frons (Figure 13); male terga reddish-yellow laterally (Figure 14b). Surstylus in Figure 17b. Similar to *M. megavidus*, from which it differs in larger size, wing morphometry, molecular data and distribution (Figure 3, Figure 4, Figure 5, Figure 6, Figure 19 and Figures S1, S3 and S4).

Description. Male.

Head. Antenna (Figure 12b) orange, basoflagellomere 1.8–2.0 times as long as wide, 2.0 times longer than pedicel, concave, with acute apex; arista pale, but dark brown in apical ⅔, and thickened basally, 1.4 times longer than basoflagellomere; covered with short, dense microtrichia. Face and frons black, covered with long orange and golden-yellow pile and silver, dense pollinose. Oral margin shiny black, except for the lateral pollinose areas (Figure 13a). Vertical triangle isosceles, shiny black except in front of the anterior ocellus that has pale pollinosity, covered with long orange-yellow pile except for black pile on the ocellar triangle. Ocellar triangle equilateral. Eye contiguity about 12 ommatidia long. Vertical triangle:eye contiguity:ocellar triangle = 1.5:0.7:1. Eye pile dense, white. Occiput with orange to yellow pile, along the eye margin with dense white pollinosity and posteriorly with metallic, bluish-greenish lustre.

Thorax. Mesonotum black with bronze lustre, covered with relatively long, dense, erect golden pile. Scutum above the wing-base usually with a patch of black pile. Scutum with two lateral and two submedian, longitudinal, white pollinose vittae. Posterior anepisternum, anteroventral and posterodorsal part of katepisternum, anepimeron, metasternum and katatergum with long golden to yellow pile and grey-green pollinose. Wing hyaline, with dense microtrichia; veins dark brown except for light brown C, Sc and R1. Calypter pale yellow. Haltere with light brown pedicel and yellow capitulum. Legs orange to yellow, except for the black metafemur and basal ¾ of the pro- and mesofemora. Pile on legs yellow to golden. Metafemur (Figure 8c) medium wide and slightly curved, about 3.6 times as long as wide. Pile on metafemur extremely short posteroventrally.

Abdomen. Dark, with a pair of white or grey, pollinose fasciate maculae, tapering, 1.4 times longer than mesonotum (including scutellum). Terga usually orange and reddish except for black tergum 1 and central parts of terga 2–4 (Figure 14b). Terga 2–4 each with a pair of white pollinose fasciate maculae (Figure 14b). Pile on terga golden-yellow. Sterna translucent, orange to brown towards the tip of the abdomen, covered with long yellow to whitish pile.

Female. Similar to the male except for typical sexual dimorphism and for the following characteristics: basoflagellomere broader and longer; frons usually with two wide (about 1/3 width of frons), lateral, silver pollinose longitudinal vittae; frons in the widest part about 0.25 width of head; white pollinose, longitudinal vittae on scutum more visible; terga usually red, except for tergum 1 and darkened parts of terga 2–4 (as in Figure 15a); white pollinose, transverse fasciate maculae present on terga 3–4 (as in Figure 15a); terga 2–3 with black pile on dark parts; white pollinose fasciate maculae solely with pale pile (as in Figure 15a).

Distribution. Greece, Samos island (Figure 19).

Etymology. The Latin adjective “magnus” (big, great) pertains to the size of the new species, larger than usual-sized specimens of related *Merodon avidus*.

*Merodon nigroscutum* Vujić, Radenković et Likov sp nov.

urn:lsid:zoobank.org:act:7A2C967A-F0F6-4E7A-B66F-74F214073CBD

Type material. Holotype: 1 ♂, pinned. Original labels: “IRAN: Mázandarán prov./3.2km S of Kandelous/36.2981528, 51.5774917/4. VI. 2015 1877m/P. Baňař lgt.”, “Potential holotype has to/be deposited in Moravian/Museum in Brno,/Czech Republic,/PetrBanar@seznam.cz”, “18296”, “WM 2960”, “TS 560”. Paratypes: IRAN: 3 ♂♂; 3.2km S of Kandelous; 36.2981528, 51.5774917; 4 June 2015; P. Banar leg.; FSUNS ID 18298, 18299, 18301 16 ♀♀; same data as for preceding; FSUNS ID 18297, 18300, 18302, 18304, 18305, 18306, 18307, 18308, 18309, 18310, 18311, 18312, 18313, 18314, 18315, 18316 1 ♂; Kelardasht, Tuydareh; 36.537973, 51.136031; 23 July 2012; Moghaddam M., Nematian M. leg.; GE coll; FSUNS ID 02335 1 ♂; Ramsar, Eshkatehchal; 36.612720, 51.288437; 25 July 2002; M. Moghaddam, M. Nematian leg.; GE coll; FSUNS ID 02336; AZERBAIJAN: 1 ♂; Avash; 38.83, 48.17; 17 June 1996; M. Hauser leg.; ZFMK (ZFMK-DIP-00069607); FSUNS ID 25340; det. M. Hauser as *Merodon avidus*.

**Figure 16:**
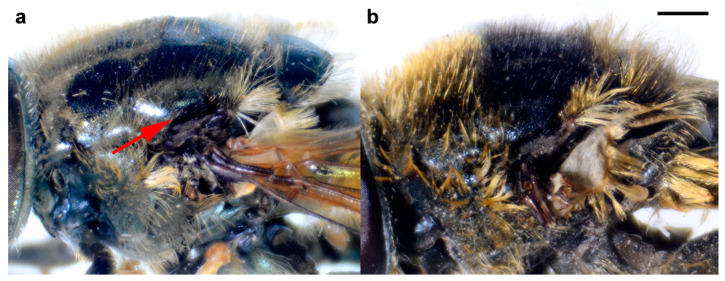
Thorax of female, lateral view. (**a**) *M. atroavidus*, (**b**) *M. nigroscutum* (scale bar 1 mm).

**Figure 17:**
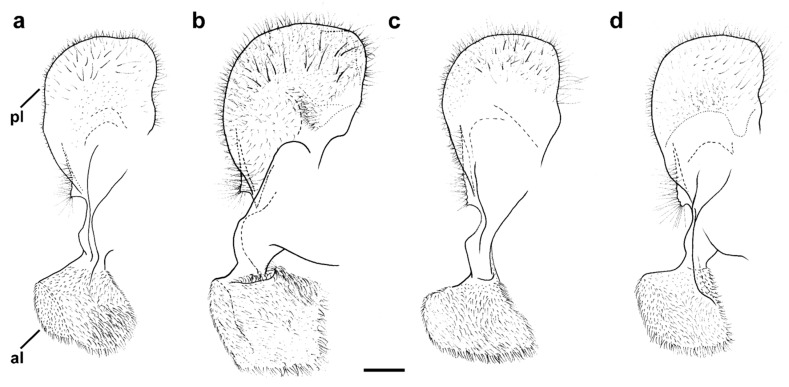
Male genitalia, surstylar lobe, lateral view. (**a**) *M. atroavidus*, (**b**) *M. magnus*, (**c**) *M. nigroscutum* and (**d**) *M. pseudomoenium* (scale bar 0.1 mm). pl—posterior surstylar lobe; al—anterior surstylar lobe.

Diagnosis. Medium sized species (11–13 mm). Terga in male black (Figure 14c), in female only tergum 2 with reddish lateral triangular maculae (Figure 15b). Posterior half of the scutum is mostly covered with black pile, except the posterior margin (Figure 16b); scutellum usually with black pile at least medially. Legs: tibiae reddish-brown, dark brown medially (Figure 8b). Basoflagellomere dark brown (Figure 12c). Surstylus in Figure 17c.

Description. Male.

Head. Antenna (Figure 12c) dark brown to black, basoflagellomere 1.8–2.0 times as long as wide, 2.0 times longer than pedicel, concave, with acute apex; arista pale, but dark brown in apical ⅔, and thickened basally, 1.4 times longer than basoflagellomere; covered with short, dense microtrichia. Face and frons black, covered with long golden-yellow pile and silver, dense pollinose. Oral margin shiny black, except for the lateral pollinose areas (as in Figure 13a). Vertical triangle isosceles, shiny black except in front of the anterior ocellus that has pale pollinosity, covered with long orange-yellow pile except for black pile on the ocellar triangle. Ocellar triangle equilateral. Eye contiguity about 12 ommatidia long. Vertical triangle:eye contiguity:ocellar triangle = 1.5:0.7:1. Eye pile dense, white. Occiput with orange to yellow pile, along the eye margin with dense white pollinosity and posteriorly with metallic, bluish-greenish lustre.

Thorax. Mesonotum black with bronze lustre, covered with relatively long, dense, erect yellowish pile anteriorly and black posteriorly; scutellum usually with black pile at least medially. Scutum with two lateral and two submedian, longitudinal, white pollinose vittae. Posterior anepisternum, anteroventral and posterodorsal part of katepisternum, anepimeron, metasternum and katatergum with long golden to yellow pile and grey-green pollinose. Wing hyaline, with dense microtrichia; veins dark brown except for light brown C, Sc and R1. Calypter pale yellow. Haltere with light brown pedicel and yellow capitulum. Legs yellow to brown, except for the black metafemur and basal ¾ of the pro- and mesofemora. Pile on legs yellow. Metafemur (Figure 8b) medium wide and slightly curved, about 3.6 times as long as wide. Pile on metafemur short posteroventrally.

Abdomen. Dark, with a pair of white or grey, pollinose fasciate maculae, tapering, 1.4 times longer than mesonotum (including scutellum). Terga black (Figure 14c). Terga 2–4 each with a pair of white pollinose fasciate maculae (exceptionally absent only on tergum 2) (Figure 14c). Pile on terga yellowish and black. Sterna translucent, orange to brown towards the tip of the abdomen, covered with long yellow to whitish pile.

Female. Similar to the male except for typical sexual dimorphism and for the following characteristics: basoflagellomere broader and longer; frons usually with two wide (about 1/3 width of frons), lateral, silver pollinose longitudinal vittae; frons in the widest part about 0.25 width of head; white pollinose, longitudinal vittae on scutum more visible; tergum 2 reddish (Figure 15b); white pollinose, transverse fasciate maculae present on terga 3–4 (Figure 15b); terga 2–3 with black pile on dark parts; white pollinose fasciate maculae solely with pale pile (Figure 15b).

**Figure 18:**
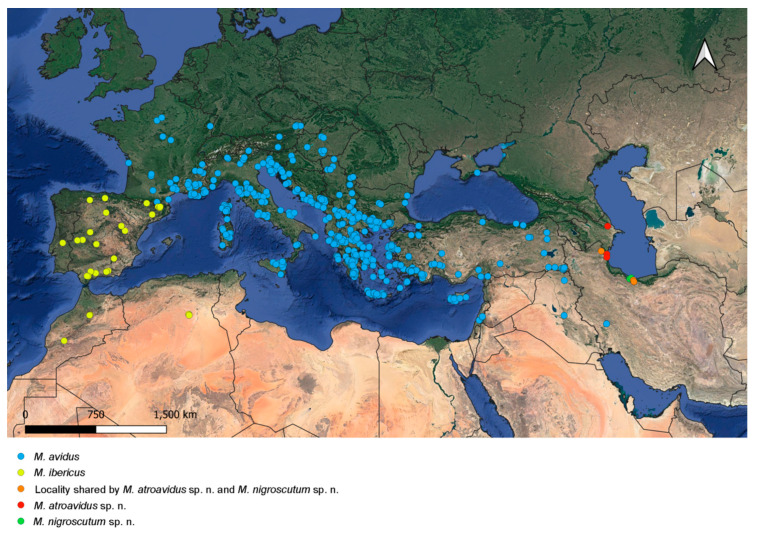
Distribution map of *M. avidus, M. ibericus, M. atroavidus* sp. nov and *M. nigroscutum* sp. nov.

Distribution. Caspian region of Iran and Azerbaijan (Figure 18).

Etymology. The name *nigroscutum* is derived from the Latin adjective “niger” (black, dark) and scutum (body part), referring to the black pilose posterior part of scutum as an important diagnostic character of this new species.

*Merodon pseudomoenium* Vujić, Kočiš Tubić et Ačanski sp nov.

urn:lsid:zoobank.org:act:586606AB-D859-40F4-B3D6-DF64B460F670

Type material. Holotype: 1 ♂, pinned. Original labels: “Greece, Corfu,/Strinilas 08/08/2014/39.739862 19.837307/Leg. Vujić”, “07795”, “TS 726”. Paratypes: GREECE: 1 ♂; Nr Ano Korakiana; 39.69882, 19.786956; 34 May 2016; A. Vujić, J. Ačanski, M. Miličić, Z. Nedeljković leg.; FSUNS ID 11447 4 ♂♂; Between Tripolis and Sparta; 37.30416, 22.4211; 23 May 2014; A. Vujić, J. Ačanski leg.; FSUNS ID06582, 06583, 06584, 06585 1 ♂; Karyes, 25 km N from Sparta; 37.30416, 22.4211; 23 May 2014; A. Vujić, J. Ačanski leg.;FSUNS ID 06551 1 ♀; same data as for preceding; FSUNS ID 06558 2 ♂♂; Karyes, 25 km N from Sparta; 37.304145, 22.421241; 22 May 2016; A. Vujić, J. Ačanski, M. Miličić, Z. Nedeljković leg.;FSUNS ID 11648, 11653 2 ♂♂; near Erimanthos 1; 38.115177, 21.772531; 22 May 2014; A. Vujić leg.; FSUNS ID 06690, 06691 2 ♂; Kambos Gorge; 36.95963131, 22.23752975; 17 May 1994; M. Ohl leg.; ZHMB; FSUNS ID 05889, 05890; det. M. Hauser as *Merodon avidus,* A. Vujić as *Merodon avidus* 1 ♂; Corfu; 39.6666667, 19.75; FSUNS ID 02323; det. Bergenst as *Merodon aberrans*, Sack P. as *Merodon aurifer* 3 ♂♂; near Falanthos; 37. 574139, 22.254096; 7 June 2017; A. Vujić, Z. Nedeljković, L. Likov, M. Miličić, T. Tot leg.; FSUNS ID 15715, 15716, 15717 1 ♀; same data as for preceding; FSUNS ID 15718 2 ♂♂; Ano Davia; 37. 542175, 22. 281499; 7 June 2017; A. Vujić, Z. Nedeljković, L. Likov, M. Miličić, T. Tot leg.; FSUNS ID 15744, 15745 1 ♀; same data as for preceding; FSUNS ID 15746 1 ♀; near Konitsa 3; 40. 050900, 20. 767013; 4 June 2017; A. Vujić, Z. Nedeljković, L. Likov, M. Miličić, T. Tot leg.; FSUNS ID 15821 3 ♂♂; Konitsa 1; 40.048864, 20. 762127; 4 June 2017; A. Vujić, Z. Nedeljković, L. Likov, M. Miličić, T. Tot leg.; FSUNS ID 15837, 15838, 15839 1 ♀; same data as for preceding; FSUNS ID 15841 1 ♀; Kastria; 37.969602, 22.142464; 6 June 2017; A. Vujić, Z. Nedeljković, L. Likov, M. Miličić, T. Tot leg.; FSUNS ID 15855 1 ♂; Near Agia Varvara; 37.937276, 22.143551; 6 June 2017; A. Vujić, Z. Nedeljković, L. Likov, M. Miličić, T. Tot leg.; FSUNS ID 15861 1 ♀; near Eleftero 1; 40.056551, 20. 850901; 5 June 2017; A. Vujić, Z. Nedeljković, L. Likov, M. Miličić, T. Tot leg.; FSUNS ID 15927 1 ♂; near Konitsa 2; 40.055502, 20.816842; 4 June 2017; A. Vujić, Z. Nedeljković, L. Likov, M. Miličić, T. Tot leg.; FSUNS ID 16056 1 ♂; Rethi; 38.015120, 22.494527; 7 September 2018; A. Vujić Z. Nedeljković, T. Tot, L. Likov, M. Janković, M. Miličić, S. Popov leg.; FSUNS ID 21078 2 ♂♂; 17 km E Igoumenitsa; 13 June 1994; A. Koch leg.; ZFMK; FSUNS ID 25331, 25332 1 ♂; Mountain Aenos; 38.115631, 20.707394; 6 July 2001; WM de Coursy leg.; CWM coll.; FSUNS ID 10370; det. Vujić A. as *Merodon avidus* 1 ♂; Parori; 37.0666667, 22.3833333; 3 May 1993; RMNH; FSUNS ID 04228 1 ♂; Platania; 39.1500, 23.3167; 24 May 2009; K. Standfuss leg.; MZH; FSUNS ID 04979; det. Standfuss K. as *Merodon avidus* 1 ♂; Mountain Mainalo; 37.674778, 22.232187; 26 May 1986; H. Teunissen leg.; RMNH; FSUNS ID 04075 1 ♀; Pigadia; 37.414284, 23.378966; June 1901; Holtz leg.; ZHMB; FSUNS ID 05896 1 ♂; Pantokrator; 39.7469444, 19.8711111; 16–30 May 1971; RMNH; FSUNS ID 04239; det. Van der Goot vs. as *Merodon avidus*, Hurkmans as *Merodon avidus* 1 ♂; Platania; 39.1500, 23.3167; 10 May 2009; K. Standfuss leg.; MZH; FSUNS ID 04984; det. Standfuss K. as *Merodon avidus* 1 ♂; Kuakiou river; 37.083, 22.317; 3 June 2000; FSUNS ID 02286; det. Vujić A. as *Merodon avidus* 1 ♂; Aegina, Chrysoleontissa 3; 37.7266, 23.4871; 15–17 June 2013; S. Magaroni leg.; MAegean (UOTA MEL 062492); FSUNS ID 07770.

**Figure 19:**
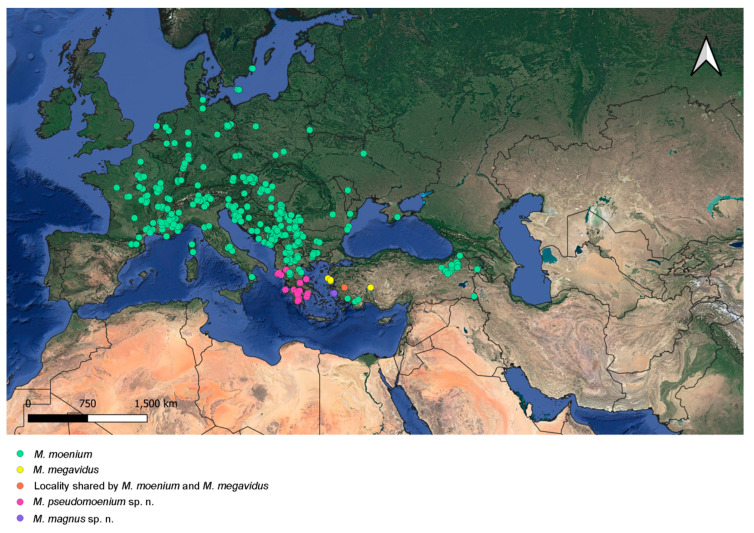
Distribution map of *M. megavidus, M. moenium, M. magnus* sp. nov and *M. pseudomoenium* sp. nov.

Diagnosis. Large to medium sized species (13–16 mm), with mostly dark terga and metatibia. Similar to *M. moenium*, from which it differs in wing morphometry, molecular data, distribution (Figure 3, Figure 4, Figure 5, Figure 6, Figure 19 and Figures S1, S3 and S4) and by the presence of pollinose fasciate maculae on tergum 2 and broader pollinose fasciate maculae on tergum 4, about 1/3 of its length (Figure 14d). Surstylus in Figure 17d.

Description. Male.

Head. Antenna dark brown to black, basoflagellomere 1.8–2.0 times as long as wide, 2.0 times longer than pedicel, concave, with acute apex; arista pale, but dark brown in apical ⅔, and thickened basally, 1.4 times longer than basoflagellomere; covered with short, dense microtrichia. Face and frons black, covered with long golden-yellow pile and silver, dense pollinose. Oral margin shiny black, except for the lateral pollinose areas (as in Figure 13a). Vertical triangle isosceles, shiny black except in front of the anterior ocellus that has pale pollinosity, covered with long orange-yellow pile except for black pile on the ocellar triangle. Ocellar triangle equilateral. Eye contiguity about 12 ommatidia long. Vertical triangle: eye contiguity: ocellar triangle = 1.5: 0.7: 1. Eye pile dense, white. Occiput with orange to yellow pile, along the eye margin with dense white pollinosity and posteriorly with metallic, bluish-greenish lustre.

Thorax. Mesonotum black with bronze lustre, covered with relatively long, dense, erect golden pile. Scutum above the wing-base with a patch of black pile or posterior half mostly black pilose. Scutum with two lateral and two submedian, longitudinal, white pollinose vittae. Posterior anepisternum, anteroventral and posterodorsal part of katepisternum, anepimeron, metasternum and katatergum with long golden to yellow pile and grey-green pollinose. Wing hyaline, with dense microtrichia; veins dark brown except for light brown C, Sc and R1. Calypter pale yellow. Haltere with light brown pedicel and yellow capitulum. Legs orange to yellow, except for the black metafemur and basal ¾ of the pro- and mesofemora. Pile on legs yellow to golden. Metafemur (Figure 8d) medium wide and slightly curved, about 3.6 times as long as wide. Pile on metafemur short posteroventrally.

Abdomen. Dark, with a pair of white or grey, pollinose fasciate maculae, tapering, 1.4 times longer than mesonotum (including scutellum). Terga black (Figure 14d). Terga 2–4 each with a pair of white pollinose fasciate maculae (exceptionally absent only on tergum 2) (Figure 14d). Pile on terga golden-yellow to partly black in some specimens. Sterna translucent, orange to brown towards the tip of the abdomen, covered with long yellow to whitish pile.

Female. Similar to the male except for typical sexual dimorphism and for the following characteristics: basoflagellomere broader and longer; frons usually with two wide (about 1/3 width of frons), lateral, silver pollinose longitudinal vittae; frons in the widest part about 0.25 width of head; white pollinose, longitudinal vittae on scutum more visible; terga partly red (at least tergum 2), except for tergum 1 and darkened parts of terga 2–4; white pollinose, transverse fasciate maculae present on terga 2–4 (as in Figure 15a); terga 2–3 with black pile on dark parts; white pollinose fasciate maculae solely with pale pile.

Distribution. Southern Greece (Figure 19).

Etymology. The prefix “pseudo-” (from Greek ψευδής, *pseudes*, “lying, false”) is used to indicate a species with a similar general appearance to another species. In this case, *Merodon pseudomoenium* sp. nov. is very similar to *M. moenium*.

*Key for typical morpho-forms of species from Merodon avidus complex*

**Figure 20:**
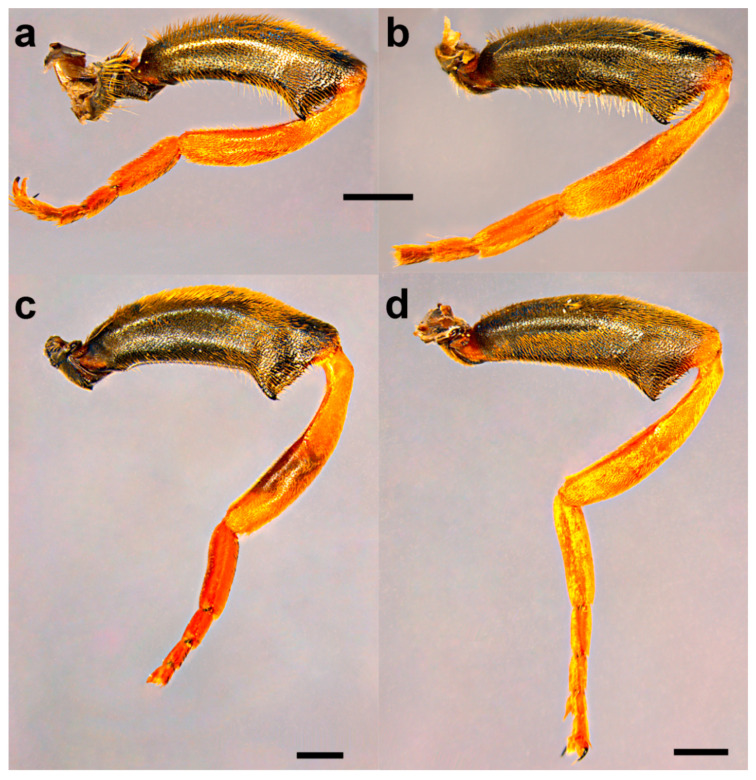
Metaleg, lateral view. (**a**,**b**) *Merodon avidus*, (**c**,**d**) *M. megavidus* ((**a**,**c**) male; (**b**,**d**) female; scale bar 1 mm).

 **1.**  Pile on metafemur very short (as in Figure 20c,d) ……….…………….…………...........2

**-**  Pile on metafemur longer (as in Figure 20a,b) ………….……………….…………...........3

**2.**  Large to medium sized species (10–16 mm); wing morphometry (Figure 5 and Figure 6), molecular data (Figure 3, Figure 4 and Figures S1, S3 and S4) and distribution: island Lesvos (Greece) and western Turkey (Figure 19) …………………….……………….…………….. *Merodon megavidus*

**-**  Large species (15–17 mm); wing morphometry (Figure 5 and Figure 6), molecular data (Figure 3, Figure 4 and Figures S1, S3 and S4) and distribution: island Samos (Greece) (Figure 19) ……………………………………………………………..………....*Merodon magnus* sp. nov.

**3.**  Terga in male black, in female only tergum 2 with reddish triangular lateral maculae (Figure 14c and Figure 15b); most of the posterior half of scutum and scutellum usually with black pilosity (Figure 16b); distribution: Caspian region of Iran and Azerbaijan (Figure 18) …………………………………………………………… ……*Merodon nigroscutum* sp. nov.

**-**  Tergum 2 in male and terga 2 and 3 in female at least partly reddish (as in Figure 1 and Figure 15a); scutum with black pile only between wing basis ……………………………………. 4

**4.**  Terga 2–4 with distinct and broad pollinose fasciate maculae (as in Figure 1a); terga reddish laterally; tibiae reddish-yellow (Figure 8a and Figure 20a,b) ……………............…...…5

**-**  Only terga 3–4 with distinct pollinose fasciate maculae, tergum 2 without or with small pollinose fasciate maculae (as in Figure 1b); only tergum 2 reddish laterally; tibiae mostly black (as in Figure 8d) …………………………………………………………………..….….6

**5.**  Antenna reddish-yellow (as in Figure 12b); wing morphometry (Figure 5 and Figure 6), molecular data (Figure 4 and Figures S3 and S4) and distribution: Europe, Turkey, Iraq and central Iran (Figure 18) ………………………….…………………………………………..*Merodon avidus*

**-**  Antenna black to dark brown (Figure 12a); wing morphometry (Figure 5 and Figure 6), molecular data (Figure 3, Figure 4 and Figures S1, S3 and S4) and distribution: Caspian region of Iran and Azerbaijan (Figure 18) ……………….………………………….……*Merodon atroavidus* sp. nov.

**6.**  Narrow pollinose fasciate maculae on tergum 4, about 1/5 of its length (Figure 1b); wing morphometry (Figure 5 and Figure 6), molecular data (Figure 4 and Figures S2–S4) and distribution: Europe and Turkey (Figure 19) …………….…………………..………... *Merodon moenium*

**-**  Broad pollinose fasciate maculae on tergum 4, about 1/3 of its length (Figure 14d); wing morphometry (Figure 5 and Figure 6), molecular data (Figure 3, Figure 4 and Figures S1, S3 and S4) and distribution: southern Greece (Figure 19) ……………….…….…*Merodon pseudomoenium* sp. nov.

### 3.5. Variability

Some specimens of species from the *Merodon avidus* complex can show morphological character variability and confuse identification. Here, we stated the extent of variability in particular species and how to overcome the potential problem in species recognition.

#### 3.5.1. *Merodon atroavidus* sp. nov.

Basoflagellomere from reddish-brown to dark brown; male terga from mostly reddish-yellow (except medially) to terga 3–4 mostly dark; black pile on scutum present on most of posterior half (except posterior margin) or only between wing bases. *Merodon atroavidus* sp. nov. can be confused with some specimens of *M. avidus*, but they do not appear sympatrically (Figure 18 and Figure 19).

#### 3.5.2. *Merodon avidus*

Adults of *M. avidus* appear in the southern part of the range of the *M. avidus* complex during most of the season, from April until October. Seasonal variability is present to a large extent. Morphological characters presented in Table 1. have gradual variations from two extreme states cited there.

Based on this variability, some specimens and populations of *Merodon avidus* can overlap with specimens of *M. atroavidus* sp. nov., *M. ibericus* and *M. moenium*. The distribution of *M. avidus* is sympatric only with *M. moenium* (Figure 18 and Figure 19). The separation of these two species at these localities can be emphasized based on molecular analyses, including a newly established diagnostic molecular marker (rRNA 28S).

#### 3.5.3. *Merodon ibericus*

Basoflagellomere from black to reddish; metatibia from mostly black to reddish-brown; male terga from all dark (except reddish lateral maculae on tergum 2) to partly reddish-yellow, at least laterally; black pile on scutum present only between wing bases, or on most of posterior half (except posterior margin). Characters of *Merodon ibericus* can overlap with *M. avidus* and *M. moenium*, but their distribution is allopatric (Figure 18 and Figure 19).

#### 3.5.4. *Merodon megavidus*

Variability in this species is found between populations from low altitudes (Vatousa, island Lesvos, 235 m a.s.l) and populations from high altitudes (Bozdağ, Turkey, 1567 m a.s.l) (Table 2).

#### 3.5.5. *Merodon moenium*

Male metatibia from mostly black to brown, in female from partly black to reddish-yellow; male terga from all dark to reddish-yellow, triangular maculae present laterally on tergum 2; black pile on scutum present between wing bases or on most of posterior half (except posterior margin); usually yellow pilose scutellum can be with black pile medially; tergum 2 without or with small pollinose fasciate maculae. Characters of *Merodon moenium* overlap with *M. pseudomoenium* and the spring generation of *M. avidus*. The distribution of *M. moenium* and *M. pseudomoenium* are allopatric (Figure 19). *Merodon moenium* is sympatric with *M. avidus* at part of its range (Figure 18 and Figure 19). The separation of these two species can be confirmed based on molecular analyses, including a newly established diagnostic molecular marker (rRNA 28S).

#### 3.5.6. *Merodon pseudomoenium* sp. nov.

Black pile on scutum present between wing bases, or on most of posterior half (except posterior margin); scutellum usually yellow pilose, but in some specimens can be with black pile medially; tergum 2 with or without small pollinose fasciate maculae. The morphological characters of *Merodon pseudomoenium* can overlap with *M. moenium*, but the distribution of the two species differs (Figure 19).

### 3.6. Distribution

Species of the *Merodon avidus* complex are distributed across continental Europe and the Mediterranean, including northern Africa (Figure 18 and Figure 19). The distribution of the newly described *M. magnus* sp. nov. is restricted to the island of Samos (Greece), *M. avidus, M. moenium* and *M. megavidus* are both continental and island species, while *M. pseudomoenium* sp. nov., *M. ibericus*, *M. nigroscutum* sp. nov. and *M. atroavidus* sp. nov. are exclusively continental. *Merodon avidus* and *M. moenium* have the widest ranges, as opposed to the newly described species that are only found in a few localities. Based on new field records, the distributional range of *M. megavidus* has now been extended to continental Turkey (the species was formerly known only from the island of Samos). Ranges of several species overlap (Figure 18 and Figure 19).

### 3.7. Biological Notes

Adults of the three closely related species with partly overlapped ranges, *Merodon avidus*, *M. moenium* and *M. pseudomoenium* sp. nov., were observed by one of the authors of this paper (Ante Vujić) in two localities with sympatric and synchronic appearance. On the island of Corfu in Greece, *M. avidus* and *M. pseudomoenium* sp. nov. were found flying together on the mountain of Pantokrator during a field trip in August 2014. The locality was composed of a few open grassland areas surrounded by maquis and Mediterranean *Quercus* forest. Adults of *M. avidus* were observed flying very fast and settling on open areas in the sun, and at the same time, adults of *M. pseudomoenium* sp. nov. were distributed near the forest, on edges mostly in the shade, flying much slower and settling on leaves.

Very similar behaviour was recorded on the island of Corsica in France in habitats along the Asco river in August 2017. This locality is situated on riverbanks with many stones along the shallow river and deciduous forest around the river. Specimens of *M. avidus* were observed settling on stones in the sun and flying very fast, while adults of *M. moenium* were distributed in the shade along the forest edge and flying much slower.

## 4. Discussion

*Merodon avidus* has intrigued taxonomists for decades. The variability of the characters of *M. avidus* has caused confusion for a long time, and the main question was whether it is a single species or a species complex. Finally, Popović et al. [27] and Ačanski et al. [22] revealed that it is a complex of closely related species by using an integrative taxonomic approach, combining information from different resources: morphology, allozymes, COI mtDNA and the geometric morphometrics of wings and surstyle of material from a broad distributional range collected during the entire season of adult activity. However, the *M. avidus* story does not end there. A more detailed examination of the material from an even wider area revealed that this intriguing group of species hid even more secrets. Here, we discover four new species for science and discuss our findings based on integrative taxonomy.

### 4.1. Molecular Evidence

Given the failure of a single COI fragment to discriminate species of the complex [22,27,28], we therefore conducted the analyses based on concatenated two COI gene fragments (5′-end and 3′-end) and introduced a new molecular marker, rRNA 28S, in the analyses of the complex. COI gene sequences delimited all newly described species (*M. atroavidus* sp. nov., *M. magnus* sp. nov., *M. nigroscutum* sp. nov. and *M. pseudomoenium* sp. nov.), as well as the earlier defined *M. ibericus* and *M. megavidus*, but failed to distinguish *M. avidus* and *M. moenium* species. Additionally, uncorrected pairwise divergence (p) of the concatenated 3′ and 5′-end of the COI gene revealed distance rates in range from 0.95% to 4.77% for new species from the *M. avidus* complex. Lower distance was observed between *M. avidus* and *M. moenium* (0.39%), while distance rates among *M. ibericus* and all other species (4.35–4.77%) revealed high divergence level. Although some detected values are lower than the suggested 2% barcoding gap, including the pairwise distances that distinguish newly described species, they are still in the range of values (0.3–2.5%) recorded for closely-related and cryptic hoverfly species [28,31,33,39,41,73].

Introducing sequences of the rRNA 28S gene in the analyses allowed us to obtain phylogenetic trees that revealed the taxonomic boundaries of all the *M. avidus* complex species, including previously undistinguished sibling species *M. avidus* and *M. moenium*. The newly described species were separated with high bootstrap support in all employed methods (BI 100, ML 98-100, MP 94-100). In the separation of the *M. avidus* and *M. moenium*, we must emphasise the obtained boundaries between these species according to the sequences of the 28S rRNA gene, which demonstrated two to three mutation positions on the constructed MP tree. Although rRNA genes are usually referred to as slowly evolving regions and represent relevant molecular markers for phylogenetic analyses of distantly related species and among taxa at higher taxonomic levels [74], the obtained data from 28S rRNA gene sequences proved to be extremely useful in the analyses of the closely related species of the *M. nanus* species group [34], as well as in the studies of the *M. aureus* species complex [45] and *M. natans* species group [2]. The study of Kočiš Tubić et al. [34] detected polymorphisms based on nuclear sequences resulting in a high level of congruence with species identification based on morphological characters. Our study results based on 28S rRNA analysis revealed clearly distinguished *M. moenium* and *M. nigroscutum* sp. nov. species as separate branches. At the same time, *M. avidus* clustered with *M. atroavidus* sp. nov. and the other analysed species shared the same haplotype.

Furthermore, including the species representing the main *Merodon* lineages following Vujić et al. [1] in our molecular analyses, we strengthened the definition of the *M. avidus* group as monophyletic. The previous publication, including a few species of the *M. avidus* group, defined these species by molecular and morphological data as a group closely related to the *M. nigritarsis* species group [25]. Molecular analyses resolved the *M. nigritarsis* and *M. avidus* groups as branches within one common lineage, the *avidus-nigritarsis* lineage [24]. The analyses conducted herein based on mtDNA COI gene sequences, as well as on the combined sequences of two genes (mtDNA COI and rRNA 28S), confirmed the species of *M. avidus* complex and *M. femoratus* species as monophyletic within the *avidus-nigritarsis* lineage with high bootstrap support (BI 100, ML 98-99). Further, within the group, *M. avidus* complex species have been distinguished as a separate clade consisting of two main branches: one corresponding to the Moroccan and Spanish samples of the *M. ibericus* species and the other to the rest of the *avidus* complex species. The two main detected branches of the species complex are in congruence with previously published studies by Milankov et al. [27], Popović et al. [28] and Ačanski et al. [22].

### 4.2. Morphological Characters

Members of the *Merodon avidus* complex can, to a great extent, be distinguished using morphological characters alone (pilosity length on metafemur, colouration of basoflagellomere, legs and terga, colour pilosity on mesonotum, pollinosity markings on terga); however, the variability of these characters must be kept in mind and they must be supplemented with other sources, such as molecular, distributional and geometric morphometric information, to be certain of valid identification.

A clear diagnostic character that separates *M. megavidus* and *M. magnus* sp. nov. from other species of the *Merodon avidus* complex is a short length of pile on metafemur, easily visible ventrally. Both species typically have orange-coloured tibiae, tarsi and basoflagellomere.

Valuable characteristics for distinguishing *M. nigroscutum* sp. nov. are the bluish-black terga in males and the presence of reddish lateral maculae only on tergum 2 in females, in addition to black pilosity at the posterior half of scutum and at least on the medial part of scutellum.

*Merodon pseudomoenium* sp. nov. is morphologically very similar to *M. moenium*, appears in similar habitats and has similar behaviour of adults. However, their distribution differs: *M. moenium* covers most of Europe, while *M. pseudomoenium* sp. nov. is present in the south of the Balkan Peninsula on the edge of *M. moenium* distribution there. This species is most probably the result of the geographic isolation of southern populations of *M. moenium* during past geological periods, potentially during glaciations. Its larger size, as well as the presence of pollinose fasciate maculae on tergum 2 and broad ones on tergum 4, helps with the proper identification of adults of these two species.

### 4.3. Geometric Morphometrics

Our analysis showed that all species had highly significantly different wing shapes. Wings are chosen as a structure for GM analysis due to their high shape heritability. Several studies on *Drosophila* have shown that environmental factors have negligible influence on wing shape [75,76,77,78]. Also, multiple studies conducted on the Syrphidae family proved that wing shape is reliable for species delimitation [2,5,13,22,35,39,40,41,79]. Regarding the *avidus* complex, Ačanski et al. [22] established species boundaries for *M. avidus*, *M. moenium*, *M. ibericus* and *M. megavidus*, using, among other things, GM of wing shape. Considering that their status has been resolved, we will focus mainly on the four newly described species, *M. atroavidus* sp. nov., *M. magnus* sp. nov., *M. nigroscutum* sp. nov. and *M. pseudomoenium* sp. nov. In both males and females, the overall percentage of correct species classification was high (males: 92.5%, females: 89.29%), showing that wings are reliable for sibling species discrimination. In the discriminant function analysis, the lowest percentage of correct classification was recorded for males of *M. atroavidus* sp. nov. and *M. nigroscutum* sp. nov., while these numbers were higher in the Bayesian classification. Also, DA cross-validation test showed a high classification rate in most species pairs. For *M. nigroscutum* sp. nov., *M. atroavidus* sp. nov. and *M. pseudomoenium* sp. nov., the classification accuracy varied when cross-validation DA was applied against different species. The correct classification rates fluctuated between 100% and 40%, with a higher frequency of higher classification rates. Pairs with fewer individual samples showed lower percentages of correct classification in cross-validation tests. This trend might be associated with the limited sample size of these pairs. However, it also suggests that these pairs could have a more similar wing shape, which could be further explored with additional material and analyses. Despite the variability in classification accuracy, the differences in wing shape among these species pairs were statistically significant and corroborated by other integrative taxonomy results. Besides significant wing shape differences, these species possess clear genetic differences, diagnostic morphological features and allopatric distribution compared with related species. It is important to emphasize that all geographically close species are separated by wing morphometry. Thus, in DA classification, no individual of *M*. *pseudomoenium* sp. nov. is classified as a *M. moenium*, and no single individuals of *M. atroavidus* sp. nov. and *M. nigroscutum* sp. nov. were classified as the other. Furthermore, it is interesting to note that not a single individual of *M. magnus* sp. nov. was classified as *M. megavidus*, which is another indication of the divergence of these two morphologically almost identical species.

### 4.4. Distribution

The distributional range of the *Merodon avidus* complex occupies Europe, the Mediterranean and the southern Caucasus region (Figure 18 and Figure 19). The *Merodon avidus* group species distribution has previously been described as mostly central- and south-European, and less Near- and Middle-Eastern and North-African [25]. However, the distribution of newly described species proves once more that the Mediterranean is—justifiably—considered a *Merodon* stronghold. The area has long been recognised as a biodiversity hotspot, owing to its geographical position and climatic and floristic conditions. Large Mediterranean peninsulas (Iberian, Balkan and Anatolian) and Greek islands are considered the most *Merodon*-rich areas [7,8,9,23], probably because the development of *Merodon* larvae is closely connected to bulbous plants—mainly Liliaceae, Amaryllidaceae and Hyacinthaceae [6,19,80,81]—which are especially numerous in the Mediterranean. In fact, the Mediterranean is a key hotspot for plants, considering that it harbours 13,000 endemics with less than 5% of the original extent of the primary vegetation [82,83]. Here we must note the important role of another European peninsula in preserving the *Merodon* species. Despite not being physically part of the Mediterranean, Crimea, specifically its southern coast, is characterised by predominantly Mediterranean vegetation and considered Sub-Mediterranean, due to its mild winters and short, dry summers [84,85]. The formation of these environmental characteristics—coined “the Mediterraneanization of Crimea”—dates back to the early Holocene when the temperatures rose and the sea level reached present-day levels [84,86]. Mediterranean climatic conditions—especially mild winters—at such high latitudes are possible due to the mountains’ protective character against the cold air from the north [87]. Within the *Merodon avidus* complex, southern Crimea represents the northernmost edge of the distribution of *M. avidus*, as opposed to *M. moenium*, which reaches further north [88] (Figure 18 and Figure 19).

Of all the studied species, *M. avidus* and *M. moenium* have the widest distributions, with the first covering most of central and southern Europe (including the Greek islands), through Turkey to Iran and Iraq, while the latter occupies most of continental Europe and the Mediterranean, almost to the Caspian Sea. The western distribution limit for both species is the Pyrenees. The mountain range acted as a geographical barrier to the expansion of *M. avidus* and *M. moenium* to the Iberian Peninsula and for *M. ibericus* eastward towards central Europe [22]. Based on new records, the distribution of *M. megavidus*, previously known only from low-altitude habitats on Lesvos island (Greece) [22], has now been extended to continental, higher-altitude habitats further to the east. The newly described *M. magnus* sp. nov., *M. atroavidus* sp. nov. and *M. nigroscutum* sp. nov. hold quite limited distributions on the island of Samos (Greece) and the southwestern Caspian region, respectively. Based on all available data collected thus far, the distribution of all three is endemic. However, this claim will be tested in the future, after more fieldwork is conducted. Taking this into account, and considering that in only the last ten years, almost 30 new species of *Merodon* have been described in the area [1,2,9,10,11,12,20,22,25,31,89], it is clear that the Mediterranean region offers fruitful ground for evolution and, subsequently, studies of hoverfly diversity.

### 4.5. Integrative Taxonomy

**Figure 21:**
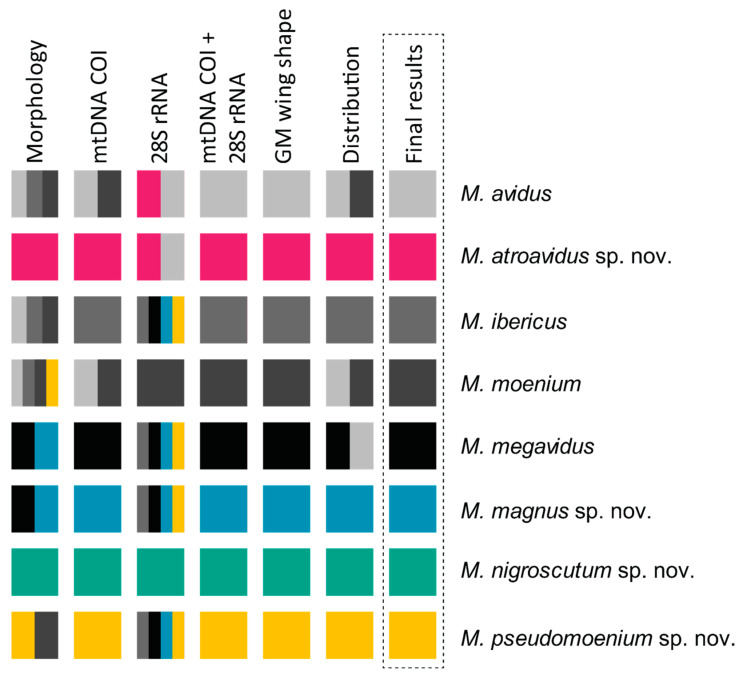
Summary of the results of integrative species delimitation. Each species is represented by a different colour. Solid colour boxes indicate successful species delimitation by a particular approach. Multicolour boxes depict clusters formed by multiple species.

Once again, an integrative taxonomy approach has shown strength in hoverfly species delimitation. Integrating results of morphology, distribution, molecular and wing shape characters revealed the presence of four new species to science (Figure 21). Two of the four new species were recognized thanks to molecular data. *Merodon magnus* sp. nov. inhabits the island of Samos and possesses extreme morphological similarity with *M. megavidus*, a species distributed on western Turkey’s mainland and the island of Lesvos from where it was described. Without the results of DNA analysis, *Merodon magnus* sp. nov. would not have been recognized as a separate species. This initial genetic data, combined with wing morphometry and subtle morphological characters, distinguished and supported recognition of this cryptic species.

The second species, *M. pseudomoenium* sp. nov., is morphologically similar to *M. moenium* and distributed in most of Europe and the Anatolian Peninsula. Without any additional evidence, small morphological differences between the two species could have been considered as intraspecific variation, especially because of the high intraspecific morphological variability of the related *M. avidus*. The separation of these two species was confirmed thanks to molecular and wing morphometric data. This distinction is also concordant with their distribution. These species are allopatric and, thus, are exposed to different environmental backgrounds that could explain their evolutionary divergence.

The distribution ranges of two species found on the western coastal mountains of the Caspian Sea, *M. atroavidus* sp. nov. and *M. nigroscutum* sp. nov., initially suggest their separate evolution. Both have morphological characters in the colouration of body pilosity that indicate the existence of independent species. However, great intraspecific morphological variability of related species from the *M. avidus* complex, especially of *M. avidus,* demanded stronger support to confirm the existence of new species. Molecular and morphometric data additionally resolved the independent position of both species. These species appear sympatric at some localities but differ in most of the analysed parameters.

In the genus *Merodon*, integrative taxonomy helped discover and confirm hidden species within different complexes. The distribution of these complexes in different lineages is variable but is now confirmed in all lineages. The *M. aureus* lineage is exceptionally rich with species complexes: *atratus* complex (resolved in [31], *aureus* and *unicolor* complexes (resolved in [45]), *caerulescens* complex (resolved in [31]), *luteomaculatus* complex [33], *chalybeus* complex (resolved in [90]), *dobrogensis* complex (resolved in [91]) and the still unresolved *sapphous* and *bessarabicus* complexes [31]. In the *albifrons* lineage, few complexes were recognized and resolved, such as the *constans* complex [13], the *equestris* complex [73], and the still unresolved *geniculatus* complex (Vujić et al., in prep.), whereas the *natans* lineage contains only the *natans* species complex (resolved in [2]), similarly to the *desuturinus* complex with the *capi* complex (partly resolved in [34]). The last two lineages include only a limited number of species (*natans*—4 and *desuturinus*—14) compared with the *albifrons* (61), *aureus* (48) and *avidus-nigritarsis* (67) lineages. Most of the species from the *avidus-nigritarsis* lineage are morphologically well-defined and, until now, without recognition of some additional species complexes, excluding the *avidus* complex. Also, the number of taxa included in the recognized complexes is usually limited from two to four, except for the *luteomaculatus* complex with six described species [33]. From this aspect, the *avidus* complex is exceptionally rich, with eight species presented here, making it the richest in the genus *Merodon*.

What is the reason for the extraordinary diversity of this complex? The answer to this question lies in species distribution and the geological history of the inhabited area. If we look at the distribution maps, we will notice that two species, *M. avidus* and *M. moenium*, are widespread, while the other species are more or less localised. Regarding these two widely distributed species, as well as *M. ibericus* and *M. megavidus*, Ačanski et al. [22] estimated their origin and diversification. According to them, diversification occurred in the Pleistocene (2.6 to 0.0117 MYA), and the first mitochondrial diversification in the *M. avidus* complex took place in the Calabrian stage of the Early Pleistocene when *M. ibericus* diverged from a common ancestor. The Günz-Mindel interglacial corresponds to the approximate period when the separation of *M. megavidus* from *M. avidus/M. moenium* lineage occurred. According to them, the most recent diversification was between *M. avidus* and *M. moenium*. Those two species colonised Europe and other areas following a postglacial “grasshopper” pattern of colonisation from Hewitt [92]. In addition to the Mediterranean geological history that unequivocally shaped the diversity of this group across the Mediterranean, it is also essential to mention the region of the southern Caspian Sea, whose geological history has shaped the diversity of the *avidus* complex in this area. Several studies have identified a region of the southern Caspian Sea as a refugium during the LGM [93,94,95,96].

## Figures and Tables

**Table 1:** Morphological variability of *Merodon avidus*.

Character	Summer	Spring
colour of basoflagellomere	reddish-yellow	dark brown
colour of male metatibia	yellow	brown medially
colour of terga	mostly reddish-yellow (except terga 1–2 medially)	mostly dark (except terga 2–3 laterally)
body pilosity	reddish	grey-yellow
pile on scutum	all yellow (except wing bases)	posterior half black pilose (except posterior margin)
pollinosity on tergum 2	distinct	indistinct

**Table 2:** Morphological variability of *Merodon megavidus*.

Character	Low Altitudes	High Altitudes
size	large (13–16 mm)	medium (10–13 mm)
metatibia	reddish-yellow	yellow with dark medial ring
body pilosity	orange to reddish-yellow	pale yellow
black adpressed pile at basitarsus of metaleg dorsally	only a few, limited to the basal half (Figure 9e)	on most of the surface (Figure 9f)

## Data Availability

The data that support this study are available in the article and accompanying online Supplementary Information. Molecular sequence data that support this study are available in a public database GenBank at https://www.ncbi.nlm.nih.gov/genbank/, accessed on 25 January 2024. Procrustes shape coordinates are available in Table S7.

## Supplementary Materials

The following supporting information can be downloaded at: https://www.mdpi.com/article/10.3390/insects15020105/s1, Figure S1: Bayesian tree based on the concatenated COI gene fragments (5′-end and 3′-end). Bayesian posterior probabilities are indicated near nodes; Figure S2: Maximum Parsimony tree based on 28S rRNA gene sequences (filles circles stand for unique changes; open circles stand for non-unique changes; bootstrap values > 50 are presented near nodes). Strict consensus tree of 3 equally parsimonious trees, L = 56, Ci = 92, Ri = 89; Figure S3: Bayesian tree based on combined COI gene fragments (5′-end and 3′-end) and 28S rRNA gene sequences. Bayesian posterior probabilities are indicated near nodes; Figure S4: Maximum Parsimony tree based on combined COI gene fragments (5′-end and 3′-end) and 28S rRNA gene sequences (filles circles stand for unique changes; open circles stand for non-unique changes; bootstrap values > 50 are presented near nodes). Strict consensus tree of 1020 equally parsimonious trees, L = 1438, Ci = 44, Ri = 69; Figure S5: Geometric morphometric analysis of the wing shape. (a) Position of male specimens in the space defined by PC1 and PC2 axes, (b) Position of male specimens in the space defined by PC3 and PC4 axes, (c) Position of female specimens in the space defined by PC1 and PC2 axes, (d) Position of male specimens in the space defined by PC3 and PC4 axes; Table S1: List of molecularly analysed samples with GenBank accession numbers (sequences newly generated herein are in boldface)and geometric morphometric analysis; Table S2: Uncorrected pairwise (p) distances between sequences of the combined 3′ and 5′ mitochondrial COI gene fragments; Table S3: Results of Principal component analysis conducted on males wing shape variables; Table S4: Results from discriminant analysis of wing shape differences among males ofinvestigated species. Above diagonal *p* values. Bellow diagonal F values. * *p*< 0.05, ** *p*< 0.01; Table S5: The results of the DA cross-validation test of the wing shape; Table S6: Results from discriminant analysis of wing shape differences among females ofinvestigated species. Above diagonal *p* values. Bellow diagonal F values. * *p*< 0.05, ** *p*< 0.01; Table S7: Procrustes shape coordinates.
