# Supplementary material for: The Extraordinary Diversity of Merodon avidus Complex (Diptera: Syrphidae)—Adding New Areas, New Species and a New Molecular Marker
Source: Insects. 2024 Feb 2;15(2):105. doi: 10.3390/insects15020105 (PMC10888622; doi:10.3390/insects15020105)
Supplement: Supplementary file 1 [file insects-15-00105-s001.zip › Supplementary Figures.pdf]

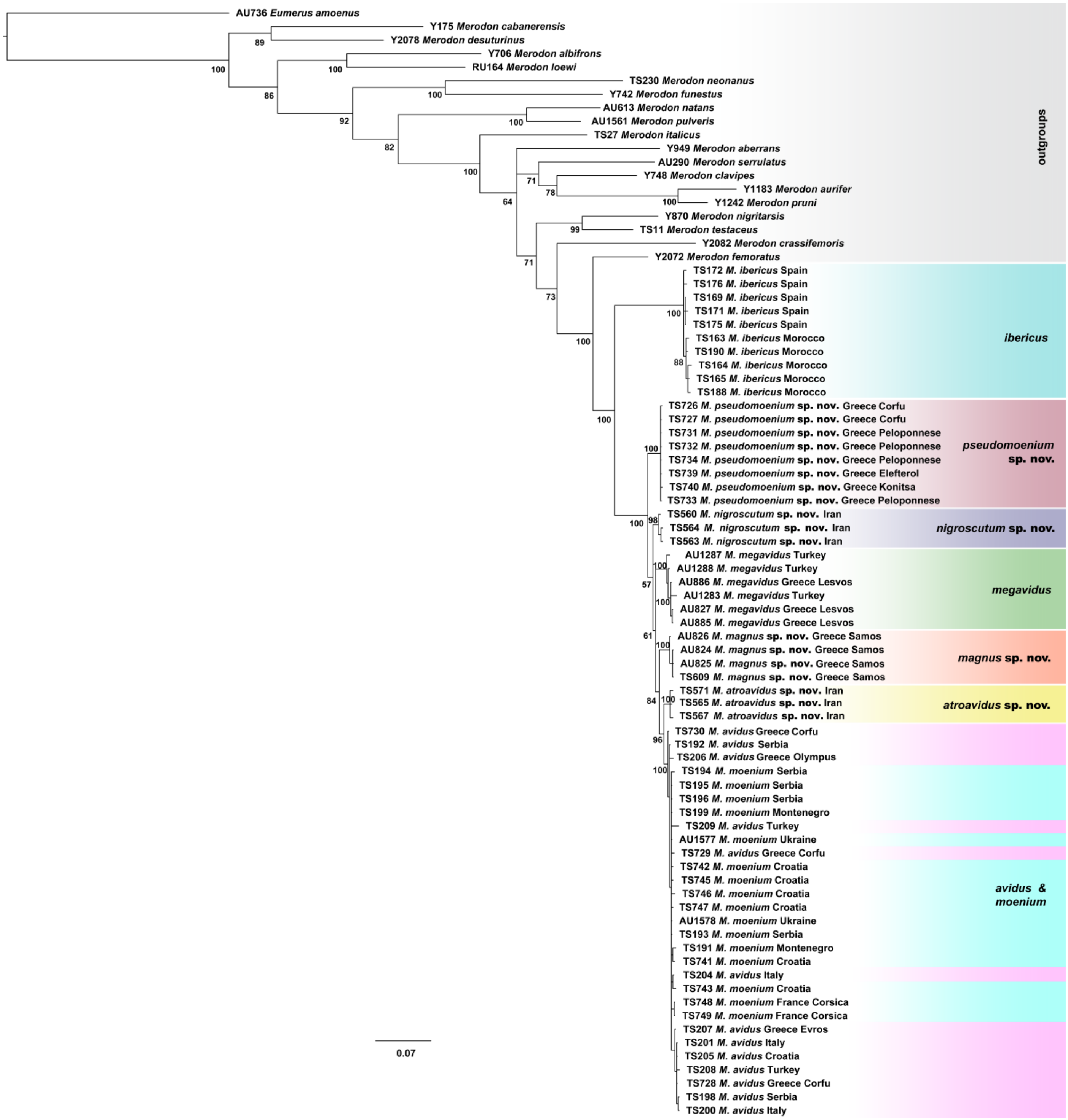

Figure S1: Bayesian tree based on the concatenated COI gene fragments (5′-end and 3′-end). Bayesian posterior probabilities are indicated near nodes.

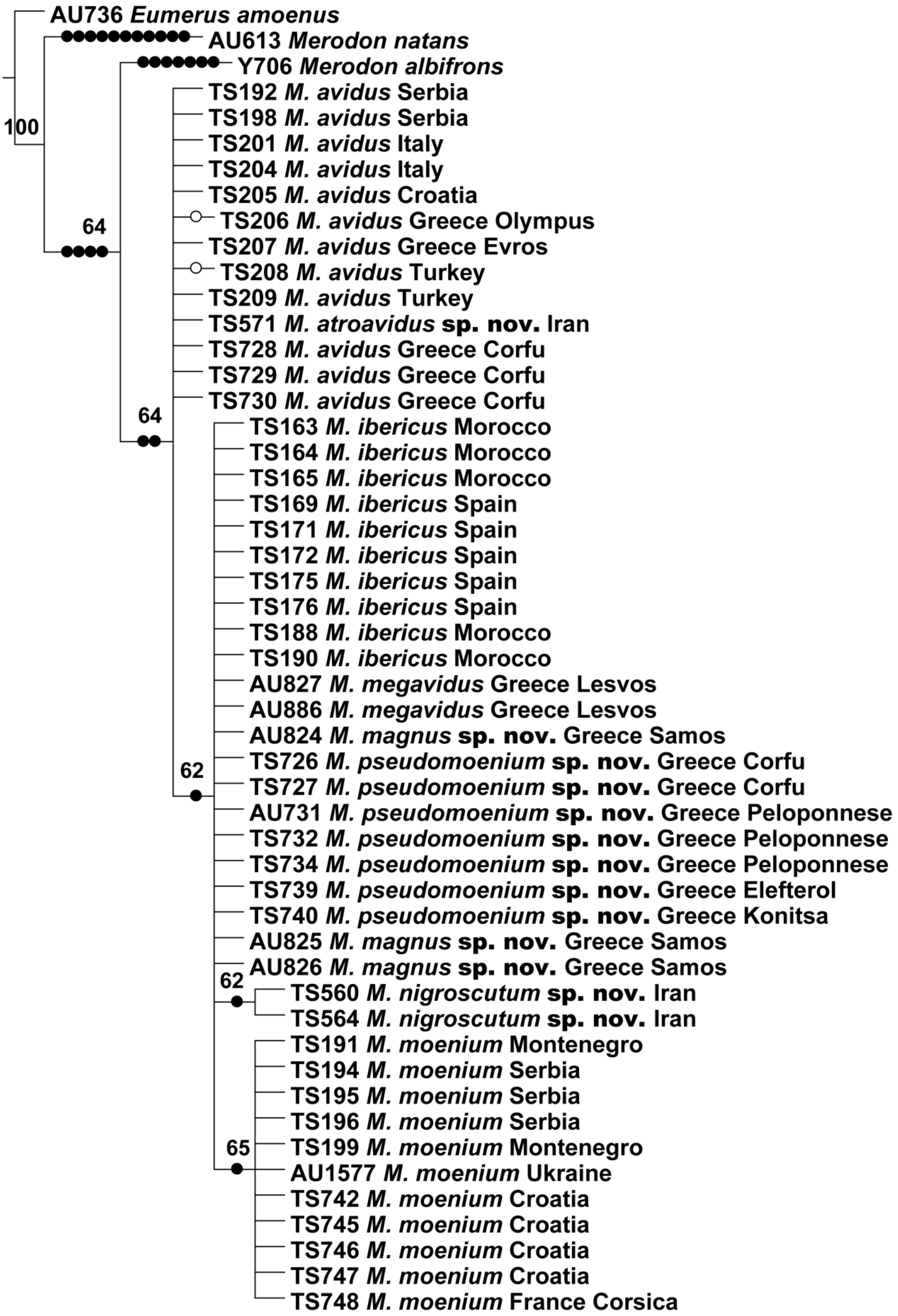

Figure S2: Maximum Parsimony tree based on 28S rRNA gene sequences (filles circles stand for unique changes; open circles stand for non-unique changes; bootstrap values > 50 are presented near nodes). Strict consensus tree of 3 equally parsimonious trees, L = 56, Ci = 92, Ri = 89.

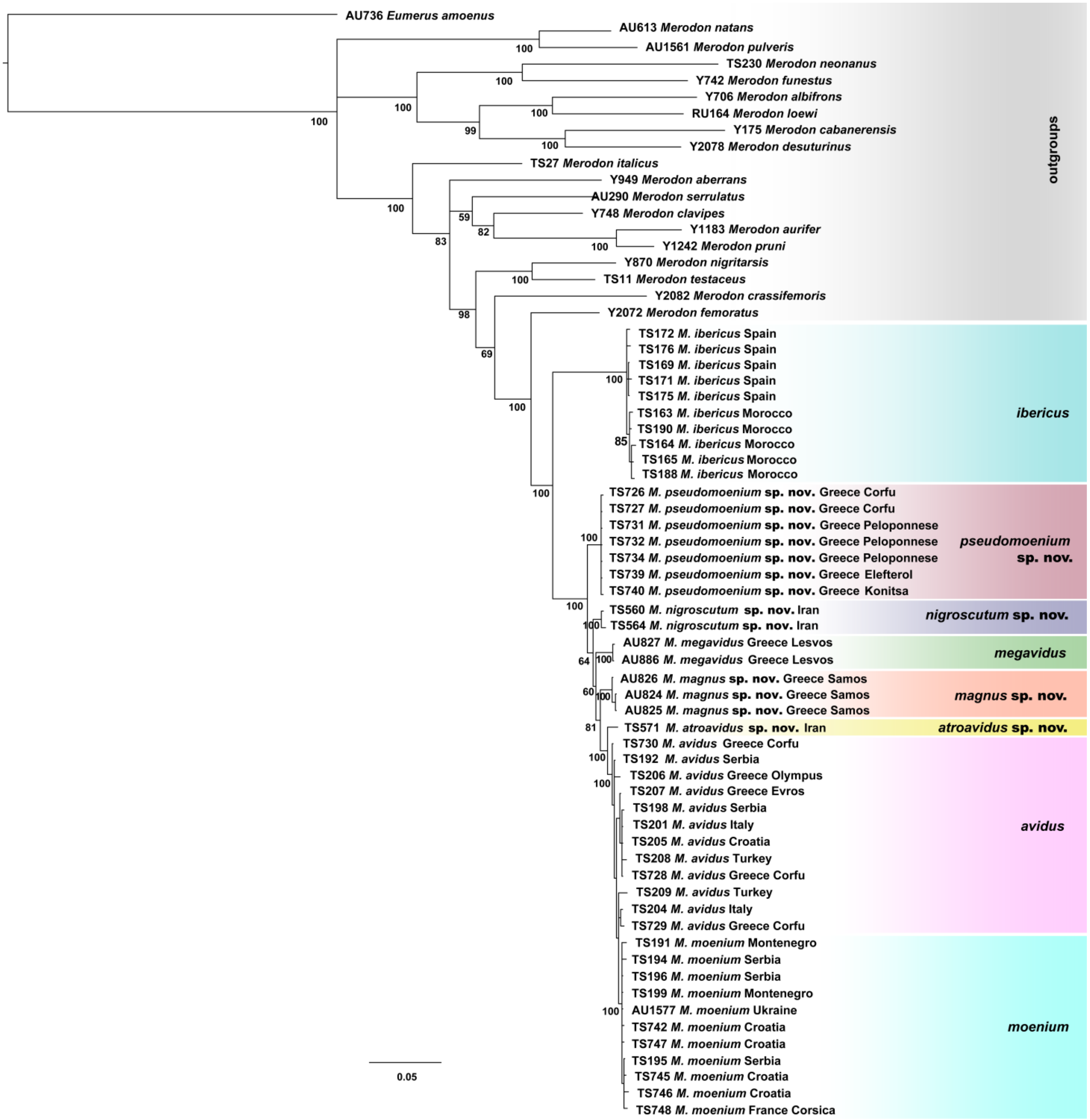

Figure S3: Bayesian tree based on combined COI gene fragments (5′-end and 3′-end) and 28S rRNA gene sequences. Bayesian posterior probabilities are indicated near nodes.

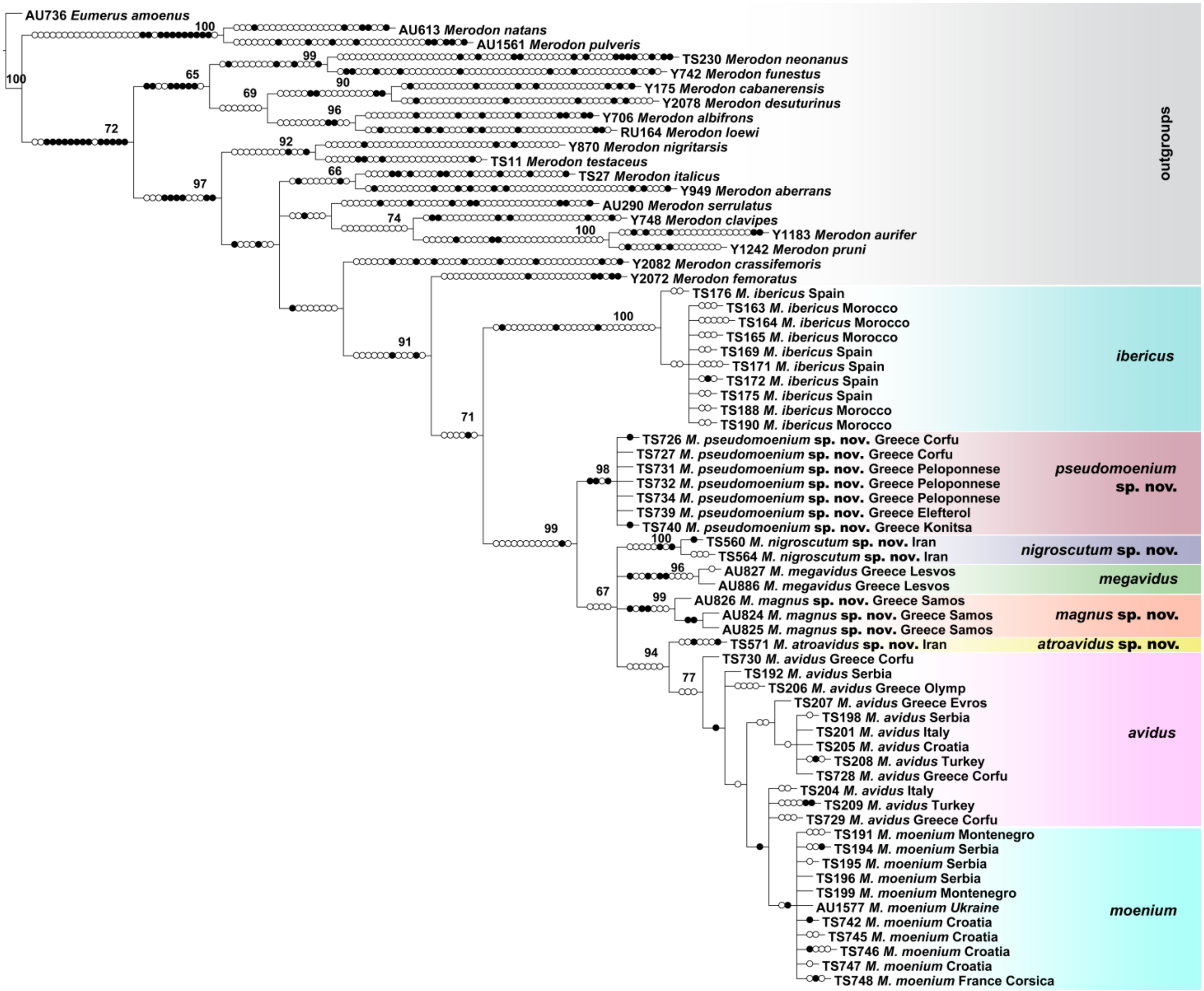

Figure S4: Maximum Parsimony tree based on combined COI gene fragments (5′-end and 3′-end) and 28S rRNA gene sequences (filles circles stand for unique changes; open circles stand for non-unique changes; bootstrap values > 50 are presented near nodes). Strict consensus tree of 1020 equally parsimonious trees, L = 1438, Ci = 44, Ri = 69.

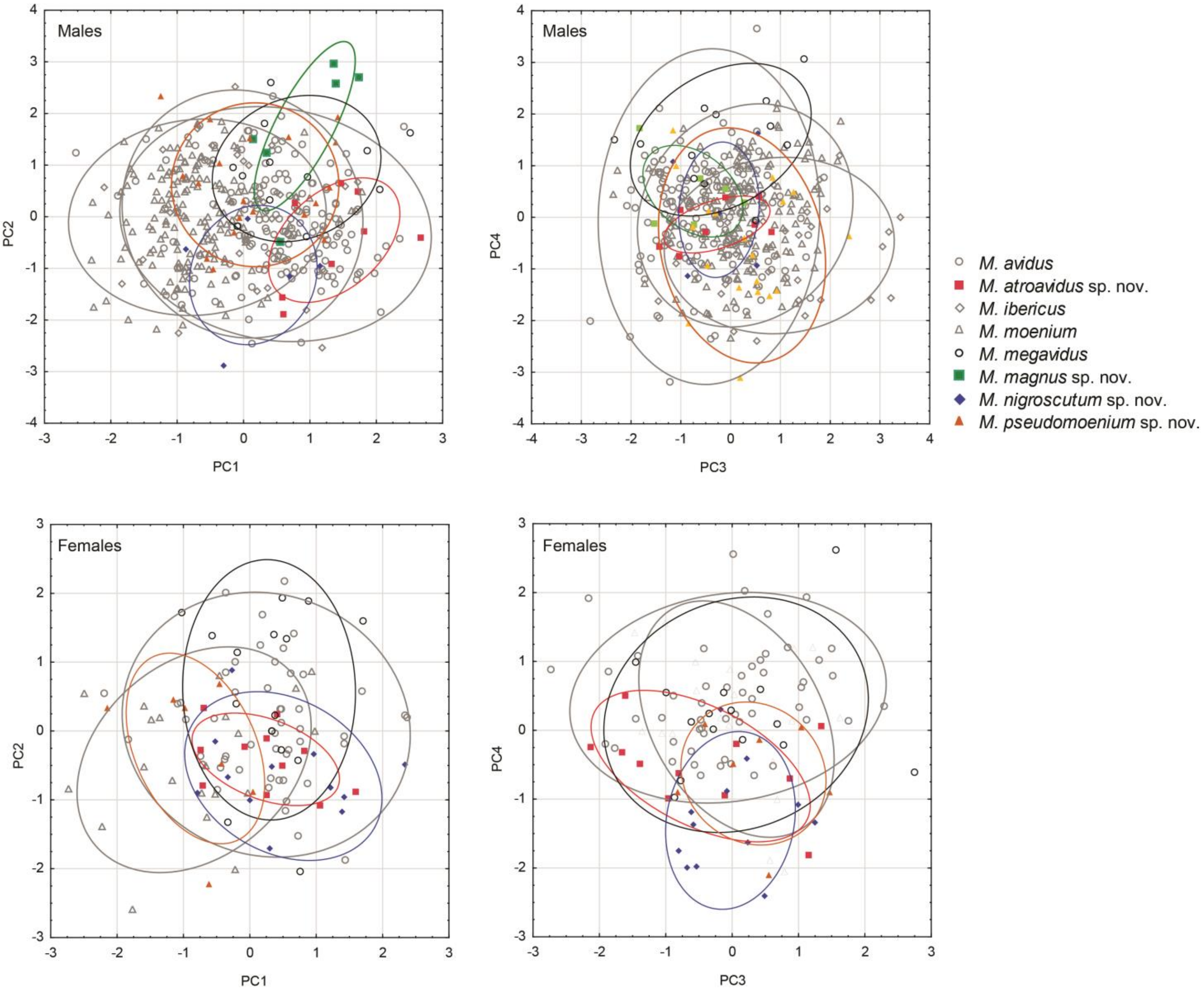

Figure S5: Geometric morphometric analysis of the wing shape. (a) Position of male specimens in the space defined by PC1 and PC2 axes, (b) Position of male specimens in the space defined by PC3 and PC4 axes, (c) Position of female specimens in the space defined by PC1 and PC2 axes, (d) Position of male specimens in the space defined by PC3 and PC4 axes;
